# A genetic engineering strategy for editing near-infrared-II fluorophores

**DOI:** 10.1038/s41467-022-30304-9

**Published:** 2022-05-23

**Authors:** Rui Tian, Xin Feng, Long Wei, Daoguo Dai, Ying Ma, Haifeng Pan, Shengxiang Ge, Lang Bai, Chaomin Ke, Yanlin Liu, Lixin Lang, Shoujun Zhu, Haitao Sun, Yanbao Yu, Xiaoyuan Chen

**Affiliations:** 1 State Key Laboratory of Molecular Vaccinology and Molecular Diagnostics & Center for Molecular Imaging and Translational Medicine School of Public Health, Xiamen University, Xiamen, 361102 China; 2 National Institute of Biomedical Imaging and Bioengineering (NIBIB), National Institutes of Health (NIH), Bethesda, MD 20892 USA; 3 State Key Laboratory of Supramolecular Structure and Materials, College of Chemistry, Jilin University Changchun, 130012 China; 4 Joint Laboratory of Opto-Functional Theranostics in Medicine and Chemistry, The First Hospital of Jilin University, Changchun, 130021 PR China; 5 State Key Laboratory of Precision Spectroscopy, School of Physics and Materials Science, East China Normal University, Shanghai, 200062 China; 6 J. Craig Venter Institute, 9714 Medical Center Drive, Rockville, MD 20850 USA; 7 Department of Chemistry and Biochemistry, University of Delaware, Newark, Delaware, 19716 USA; 8 Departments of Diagnostic Radiology, Surgery, Chemical and Biomolecular Engineering, Biomedical Engineering, Yong Loo Lin School of Medicine and Faculty of Engineering, National University of Singapore, 117597 Singapore, Singapore; 9 Clinical Imaging Research Centre, Centre for Translational Medicine, Yong Loo Lin School of Medicine, National University of Singapore, Singapore, 117599 Singapore; 10 Nanomedicine Translational Research Program, NUS Center for Nanomedicine, Yong Loo Lin School of Medicine, National University of Singapore, Singapore, 117609 Singapore

**Keywords:** Pharmacokinetics, Fluorescence imaging

## Abstract

The second near-infrared (NIR-II) window is a fundamental modality for deep-tissue in vivo imaging. However, it is challenging to synthesize NIR-II probes with high quantum yields (QYs), good biocompatibility, satisfactory pharmacokinetics, and tunable biological properties. Conventional long-wavelength probes, such as inorganic probes (which often contain heavy metal atoms in their scaffolds) and organic dyes (which contain large π-conjugated groups), exhibit poor biosafety, low QYs, and/or uncontrollable pharmacokinetic properties. Herein, we present a bioengineering strategy that can replace the conventional chemical synthesis methods for generating NIR-II contrast agents. We use a genetic engineering technique to obtain a series of albumin fragments and recombinant proteins containing one or multiple domains that form covalent bonds with chloro-containing cyanine dyes. These albumin variants protect the inserted dyes and remarkably enhance their brightness. The albumin variants can also be genetically edited to develop size-tunable complexes with precisely tailored pharmacokinetics. The proteins can also be conjugated to biofunctional molecules without impacting the complexed dyes. This combination of albumin mutants and clinically-used cyanine dyes can help widen the clinical application prospects of NIR-II fluorophores.

## Abstract (web summary)

It is currently difficult to synthesise NIR-II probes with good quantum yields, biocompatibility and pharmacokinetics. Here the authors report a strategy to alter these properties by modifying the protein coatings with biofunctional molecules, and generate long-wavelength fluorophores for in vivo imaging.

## Introduction

Near-infrared II (NIR-II) fluorescence imaging is preferrable to near-infrared I (NIR-I) imaging as this window provides reduced tissue scattering and autofluorescence, thus achieving deeper tissue penetration with superior contrast and resolution^1–7^. The development of high-quality NIR-II fluorophores is essential to realizing deep bioimaging^1,5,8–11^ and facilitating clinical translation. For example, clinically used cyanine dyes^12–14^ were discovered to possess a tail peak in the NIR-II window when viewed using an InGaAs camera^15–20^. However, the majority of organic NIR-II fluorophores exhibit very low fluorescence quantum yields (QYs)^19^ and/or poor pharmacokinetic properties. Moreover, multiple and complex synthetic steps are followed to synthesize these fluorophores^21,22^. The photoluminescence of small molecules is controlled by their energy gap, which depends on their chemical structure through π-conjugated groups^16,21–23^. Hence, long-wavelength fluorophores bear large π-conjugated groups (*i.e*., extended photon-conjugated skeletal structures containing fused heteroaromatic rings and/or electron-withdrawing groups)^23–26^. However, fluorophores with large π-conjugated groups usually have inherently low QYs and poor pharmacokinetics^18^.

Large π-bridges can potentially induce nonradiative decay through vibrational relaxation, collisional quenching, and/or π-π stacking-induced quenching. Molecular simulation studies revealed that the low QYs exhibited by fluorophores containing large π-bridges could be attributed to the properties of the excited states of the π-bridges. The excited states can be directly attacked and subsequently quenched by water molecules and they are also prone to oxidation^3^. Additionally, large conjugated fluorophores are usually hydrophobic and do not exhibit satisfying pharmacokinetic properties^22^. In recent years, efforts have been made to synthesize rigid structures bearing electron-withdrawing groups at the center of the scaffold and/or to introduce shielding groups to reduce collisional quenching^26–29^. For example, rigid cyclohexanol structures have been introduced at the center of cyanine/polymethine dyes to produce improved dye variants. Researchers introduced a rigid alkyl thiophene moiety to increase the dihedral angle in a donor-acceptor-donor (D-A-D) dye to improve QYs^19^. Shielding units containing long polyethylene glycol (PEG) groups have also been used to develop good-performance D-A-D dyes and reduce collisional quenching. Some researchers have introduced dialkoxy-substituted benzene- and/or fluorene-based groups as the shielding units (shielding group, S) to develop S-D-A-D-S fluorophores that exhibit relatively high QYs. By modifying the PEG and S groups, the dispersion of these molecules in solution can be improved and nonradiative decay caused by collisional quenching/π-π stacking can be reduced^2,30,31^.

**Fig. 1:**
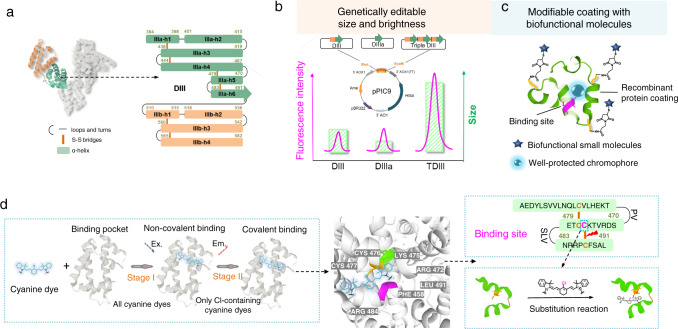
Schematic representation of the complex design and binding mechanism. **a** Left: Structure of HSA (Protein Data Bank ID: 1E78). DIIIa is highlighted in green and DIIIb is highlighted in orange. Right: Split α-helical (h) structures of DIIIa (green) and DIIIb (orange). **b** Wild-type albumin can be genetically engineered to produce fragments and recombinant proteins that bind to cyanine dyes and produce complexes with tunable size and brightness. The recombinant plasmid, DIII, DIIIa (the minimal functional unit for chaperoning cyanine dyes), and TDIII (a tandem of three DIII sequences), were inserted into the vector (*Pichia pastoris*: pPIC9 vector). **c** The chromophore coatings can be biologically or chemically modified with biofunctional molecules. **d** Mechanism of interaction between cyanine dyes and the albumin variants. Left: Two independent stages are involved in the interaction process. First, cyanine dyes insert themselves into the hydrophobic protein pocket and are tightly bound through non-covalent bonds (stage I). Second, Cl-containing dyes form a covalent bond with a specific residue of the protein (stage II). Center: 3D structure of the exact binding site for Cl-containing cyanine dyes in DIII. The colors green (Lys475), purple (Phe458), and grey (Arg472 and Arg484) represent the residues involved in the synergistic supramolecular interactions of stage I. Right: Cys476 in the binding pocket was proven to be the binding residue.

However, these conventional strategies to improve the performance of large conjugated fluorophores are based on chemical modification processes^7^ that can potentially influence the QY of the dyes. Moreover, some cyanine/polymethine dyes, such as the clinically used indocyanine green (ICG), do not possess modifiable units in their scaffolds and so cannot be easily conjugated to other moieties. Most importantly, achieving a good-performance fluorophore with high QY, biosafety, clinical translation potential, and satisfying pharmacokinetic properties as well as terrific biofunction is extremely challenging. We have previously reported that cyanine dyes can “hitchhike” with serum albumin in vivo to produce ultra-bright NIR-II fluorophores with improved pharmacokinetics^16^. The cyanine dyes inserted into a hydrophobic pocket of albumin, which stabilized the dyes and so reduced nonradiative decay and synergistically increased the extent of π-conjugation. The protein shell also protected the dyes from collisional quenching and provided biocompatibility. Inspired by this work, here we screen high-affinity recombinant subunits of albumin that could be used to generate a series of editable protein shells to chaperone dyes. Using yeast to express fragments of albumin, we successfully identify the exact binding domain for cyanine dyes. This allows us to re-engineer a series of minimal subunit-incorporating albumin variants. We use this genetic engineering technique to tune the size and brightness of cyanine/polymethine dye@protein complexes (Fig. 1a, b). Moreover, we are able to modify the protein coatings with biofunctional molecules through bioengineering techniques or chemical modification, enabling generation of long-wavelength fluorophores with biofunctional properties for in vivo imaging (Fig. 1c). This shows that a genetic engineering strategy can be used to tune the brightness, pharmacokinetic properties, and biological functions of dye molecules through tailoring high-affinity coatings instead of modifying the dyes themselves. This work ultimately provides a synthesis strategy and a set of ultra-bright NIR-II agents for deep-tissue, high-contrast intravital molecular bioimaging.

## Results

### Discovery of an albumin subunit that spontaneously binds cyanine dyes and enhances their brightness

**Fig. 2:**
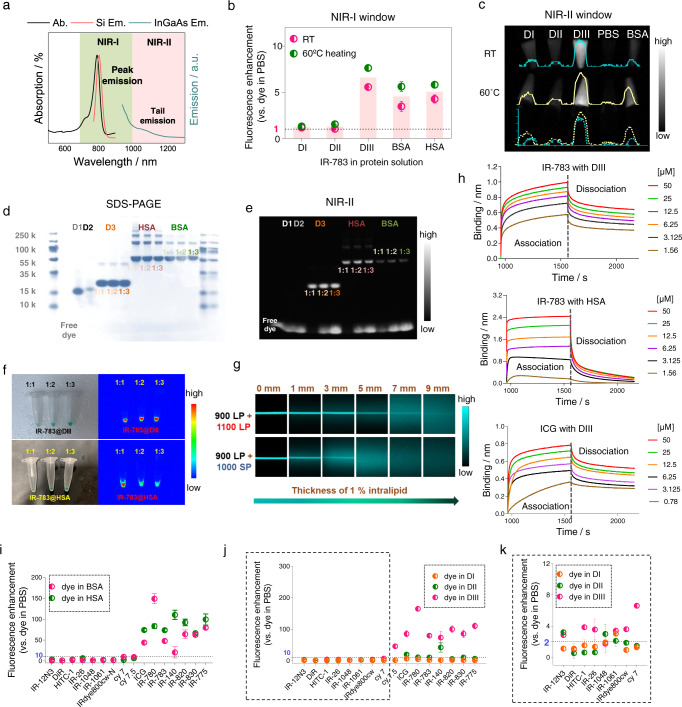
Fluorescence enhancement of cyanine dyes complexed with DIII. **a** Absorption, NIR-I emission, and NIR-II emission spectra of IR-783@DIII. Ab.: absorption spectrum; Si Em.: emission spectrum recorded using a silicon camera; InGaAs Em.: emission spectrum recorded using an InGaAs camera. **b**, **c** Fluorescence enhancement of IR-783@DIII (1:1) at room temperature (RT) and 60 °C in the NIR-I (**b**) and NIR-II (**c**) regions (*n* = 3). **d**, **e** Electrophoresis analysis of mixtures of IR-783 with DI, DII, DIII, HSA, and BSA (10 min post-heating at 60 °C) under white light (**d**) and ˃1100 nm fluorescence (**e**) imaging. **f** NIR-II images of IR-783@DIII and IR-783@HSA with various protein-to-dye molar ratios. **g** Fluorescence images of capillaries filled with IR-783@DIII solution immersed in 1% intralipid to varying depths. **h** Kinetic binding assay results of IR-783 with DIII (top) or HSA (middle) and ICG with DIII (bottom). **i**–**k** Fluorescence enhancement of various cyanine dyes complexed with BSA or HSA (**i**) and DI, DII, or DIII (**j**) compared with free dye in PBS. The results for the probes outlined with a dashed line in (**j**) are enlarged in (**k**) (*n* = 3). Data are presented as mean ± s.d. of three independent experiments.

We first screened recombinant subdomains of human serum albumin (HSA) to identify the exact binding domain for cyanine dyes. HSA consists of three independent subdomains that contain highly homologous sequences: domain I (DI), domain II (DII), and domain III (DIII)^32,33^ (Supplementary Fig. 1a). The domains were mixed with IR-783, a commercial cyanine dye, and the mixtures were compared with free dye in phosphate-buffered saline (PBS). The brightness of IR-783 increased in the NIR-I (peak emission at ~810 nm) and NIR-II (tail emission) regions only when the dye was mixed with DIII and not DI or DII (Fig. 2a–c, Supplementary Fig. 1b). Surprisingly, this fluorescence enhancement with DIII was ~1.6-fold higher than that observed for a mixture of HSA and IR-783 (Fig. 2b, c). These results indicated that DIII is the probable IR-783-binding pocket. Electrophoresis (SDS-PAGE) experiments identifying dye@protein complexes showed consistent results (Fig. 2d, e). When IR-783 was mixed with DIII, HSA, or bovine serum albumin (BSA) at a protein-to-dye molar ratio of 1:1, bands corresponding to free dye were nearly not observed. The NIR-II fluorescence intensity of bands corresponding to free dye increased as the dye loading was increased from 1:1 to 1:2 and 1:3 (Fig. 2e). At all molar ratios of DIII and IR-783 studied, bands corresponding to IR-783@DIII exhibited comparable NIR-II fluorescence intensities; in contrast, when the molar concentration of the dye was doubled (or tripled) in mixtures with HSA, the fluorescence intensities of IR-783@HSA bands decreased (Fig. 2f). These results indicated that high dye loadings induced fluorescence quenching in IR-783@HSA complexes but not in IR-783@DIII complexes. The fluorescence imaging depth of IR-783@DIII in scattering media was evaluated using a capillary phantom immersed in 1% intralipid. Deeper imaging of IR-783@DIII was possible in the NIR-II window compared with the NIR-I window (Fig. 2g). We further compared the reaction efficiencies of albumin domains DIII and DI (binding domain and nonbinding domain) at two temperatures with 10 min-incubation and 2 h-incubation, respectively. As we expected, the binding was faster at 60 °C compared to that at 37 °C, while there was no significant difference of fluorescence enhancement over both incubation time (Supplementary Fig. 2). Moreover, the optical properties were further systematically evaluated and the results demonstrated that dye@albumin/its variants exhibited great optical efficacies (Supplementary table 1, Supplementary Figs. 3 and 4).

Next, we systematically studied a series of cyanine/polymethine dyes to identify dyes that tightly bind albumin (Supplementary Fig. 5). We first performed bio-layer interferometry (BLI) experiments to evaluate the binding affinity between DIII or HSA and IR-783. IR-783 showed stronger binding affinity to DIII (*K*_d_ = 0.57 nM) than HSA (*K*_d_ = 1.7 nM) (Fig. 2h). IR-783 also showed faster association (*K*_on_ = 4.09 × 10^−4^ M^−1^ s^−1^) and slower dissociation (*K*_off_ = 0.1 s^−1^) with DIII compared to HSA (*K*_on_ = 2.51 × 10^5^ M^−1^ s^−1^, *K*_off_ = 0.2 s^−1^) (Supplementary Tables 2, 3). We next studied other cyanine/polymethine dyes including ICG, IR-780, IR-820, IR-830, IRdye800cw, Cy7, Cy7.5, IR-140, IR-1048, IR-1061, IR-12, and DiR, etc. We found that almost all of the dyes showed high binding affinities to albumin (Supplementary Tables 2–4) with *K*_d_ values in the nanomolar range, excluding DiR with a *K*_d_ value of 144 µM. Therefore, the cyanine/polymethine dyes showed high binding affinity to albumin and its binding subunit-incorporating mutant. The fluorescence intensities of ICG, IR-780, IR-783, IR-820, IR-830, IR-775, and IR-140 were significantly higher in BSA and HSA than in PBS (Fig. 2i). We further measured the brightness of the dyes in DI, DII, and DIII to confirm if the enhancement could be attributed to DIII (Fig. 2j, k). The results confirmed that DIII is the exact binding domain for cyanine/polymethine dyes. Binding to the DIII domain enhanced the brightness of most of the cyanine dyes. The remainder of the dyes (e.g., IR-12, DiR, IR-1048, IR-1061, IRdye800cw, Cy7) showed marginal fluorescence enhancement (2–8 fold) when mixed with albumin or the albumin mutants. These results indicated that the binding pose (e.g., the binding mode of the π-π conjugation group) rather than high binding affinity contributed to the brightness enhancement.

### Chloro (Cl)-containing cyanine dyes form a spontaneous covalent bond with DIII

**Fig. 3:**
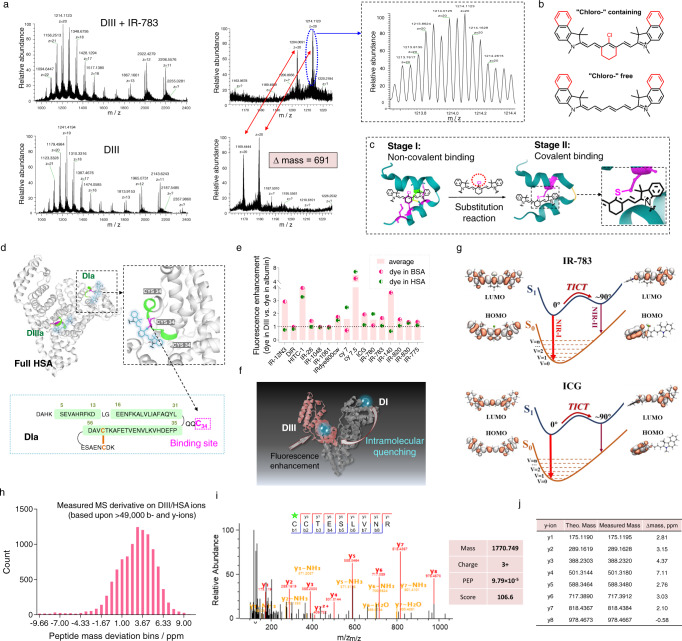
Mechanism of interaction between cyanine dyes and DIII. **a** UHPLC-MS analysis revealed the formation of a covalent bond between DIII and the inserted cyanine dye. The increase in mass was 691 m/z, which was equal to IR-783 (Cl¯ removed). **b** Core structures of Cl-containing and Cl-free dyes. Some cyanine/polymethine dyes do not contain the red group(s). **c** Cyanine dyes interact with DIII and its variants in two independent stages. In stage I, the dyes insert into the hydrophobic pocket of DIII and bind to the pocket through non-covalent bonds, resulting in increased brightness. In stage II, covalent bonds stabilize Cl-containing dyes in the pocket. The amino acid residues buried inside the pocket also play essential roles in covalent bond formation. **d** Full HSA bears two binding sites for cyanine dyes: one site is situated on the surface of DIa (free Cys34 is the only natural cystine containing a free thiol group) and the other is located in the hydrophobic pocket of DIIIa (covalent bonds are formed at Cys475). Lower panel: binding residue in the sequence. **e** Enhanced fluorescence intensity (1.3–5.9-fold) was recorded for most of the cyanine dyes bound to DIII compared with HSA or BSA. **f** The fluorescence enhancement is lower for dye@HSA than dye@DIII because the dye bound to DI exhibits intramolecular quenching to the fluorescently enhanced dye bound in the hydrophobic pocket of DIII. **g** TICT-induced NIR-I and NIR-II states of IR-783 (top) and ICG (bottom) based on DFT and TDDFT calculations. **h**–**j** Shotgun proteomics analysis revealed that Cys475 contributes to the formation of the covalent bond. MS/MS spectra (**i**) and fragment masses (**j**) of the triply charged tryptic peptide containing the dye-modified cysteine (*indicated) amino acid. The complete y series ions (y1–y8) were acquired using an Orbitrap-based mass analyzer (<0.5 ppm). These ions are labeled on the spectrum and depicted on the sequence (upper panel). The data were analyzed using MaxQuant. Posterior error probability (PEP) and Andromeda scores are presented in the right panel. Source data are provided as a Source Data file.

The binding behavior and loading capacity of cyanine dyes and albumin were further studied by ultra-high performance liquid chromatography–mass spectrometry (UHPLC-MS). Each DIII molecule bound only one IR-783 molecule (Supplementary Fig. 6). Unexpectedly, the binding was not through a non-covalent adhesion as we previously assumed, but through a covalent bond involving substitution of a Cl– group (Fig. 3a, Supplementary Fig. 7). Similar results were obtained when the experiments were conducted with other Cl-containing/free cyanine dyes such as IR-780, IR-820, and IR-830 (Supplementary Fig. 7a–c). Hence, we hypothesized that Cl-free cyanine dyes (e.g., ICG, Cy7, Cy7.5) could potentially bind to albumin and its DIII-containing mutants through non-covalent bond(s). This hypothesis was validated by the experimental results (Supplementary Fig. 7d). Collectively, these results indicated that the Cl-containing cyanine dyes bound DIII through covalent bonds, while the Cl-free dyes bound DIII through non-covalent supramolecular interactions (Fig. 3b, c). We further evaluated a different meso-halogen substituent based cyanine dye, meso-bromine cyanine dye (Br-containing dye), and received the similar binding capacity as Cl-containing dye and comparable brightness enhancement (Supplementary Fig. 8).

### Full HSA covalently binds two Cl-containing cyanine dyes and induces intramolecular quenching

The complex formed between full HSA and cyanine dyes was next studied. It was observed that one HSA molecule efficiently bound two IR-783 molecules (Supplementary Fig. 9). The results indicated that there are two independent sites for dye binding in the full HSA domain. One of the binding sites, which does not contribute to the enhancement in fluorescence intensity, is located at the free Cys34 on the surface of the subdomain DIa, which is the only cysteine molecule that bears a free thiol group in the scaffold^32^. The other binding site, which resembles the dye binding site on DIII, is a hydrophobic pocket in the subdomain DIIIa (Fig. 3d). Since these binding sites are spatially close to each other, we believe that the fluorescence quenching observed at high dye loadings in Fig. 2e, f could be attributed to intramolecular quenching (Fig. 3f). To verify our hypothesis, we used Cl-free ICG to interact with HSA and DIII since it would not bind Cys34 of HSA. Additionally, the dye bound at Cys34 on DIa would have an unrestricted conformation, which would induce additional nonradiative decay through intramolecular vibrational/rotational relaxation. As we assumed, when the molar concentration of ICG was doubled (or tripled) in mixtures with HSA, the fluorescence intensities of ICG@HSA bands were comparable as that of ICG@DIII (Supplementary Fig. 10). Collectively, these results indicated that the fluorescence intensity of Cl-containing dyes had intramolecular quenching via binding with two albumin subdomains when loading dyes increased. This assumption was also confirmed in the following section by chemoproteomics analysis. The universality of cyanine dye quenching with HSA was further determined. Figure 3e confirmed that the fluorescence increase for a series of cyanine dyes was higher when the dyes were mixed with DIII than HSA or BSA (1.3–5.9-fold higher on average). In addition, density functional theory (DFT) and time-dependent DFT (TDDFT) calculations demonstrated that IR-783 and ICG exhibited comparable twisted intramolecular charge transfer (TICT) (Fig. 3g), indicating that the fluorescence enhancement could not be attributed to the formation of covalent bonds. These results agreed with the results presented in Fig. 2 obtained from analyzing the binding affinities of cyanine dyes with albumin and the brightness of the resulting complexes.

### Shotgun proteomics technique for studying the reaction mechanism between albumin and cyanine dyes

We used an unbiased shotgun proteomics method to identify sites of nucleophilic reactions between protein residue(s) and Cl–C groups and to understand the reaction mechanism (Supplementary Fig. 11a)^34–39^. Mixtures of DIII or HSA and IR-783 were digested by four different digestion enzymes. Specific cleavage sites were cut during this process (Supplementary Fig. 11b) and the resulting peptides were analyzed using the superhigh-resolution liquid chromatography with tandem mass spectrometry (LC-MS/MS). All the peptides were screened in an unbiased manner (e.g., the top 10 ions in each full MS scan were subjected to an MS/MS scan). Following this analysis, we searched the UniProt human database (~23,000 sequences) with dye labeling as a variable modification (C_38_H_46_N_2_O_6_S_2_, mass = 690.2797 m/z). The results indicated that the dye-labeled peptide sequence was C(dye)C(carbamidomethylation)TESLVNR. The b and y ion series were further analyzed to identify the exact binding residue. The full mass (MS1) of the peptide was calculated to be 1770.749 m/z (measurement accuracy < 5 ppm). The measurement accuracy for the fragments was < 10 ppm (y ion series; y1–y8). The results revealed that the Cys476 residue was the potential binding site (Fig. 3h–j, Supplementary Figs. 12–14). The 3D crystal structures of HSA and BSA revealed that the DIIIa domain contains two disulfide bonds, one of which is formed between the Cys476 and Cys487 residues. We observed that the Cl–C group in Cl-containing cyanine dyes could react with the thiol (SH–) group in Cys476 by a nucleophilic substitution reaction. We hypothesized that during the process of peptide folding into well-defined secondary structures, Cys476 and Cys487 partially or unsuccessfully form a disulfide bond. Therefore, free SH– groups would be present in the hydrophobic pocket or Cys476 would be exposed in an attackable environment. The inequivalence of Cys476 and Cys487 residues in dye binding could be attributed to the proximity of the binding sites achieved post non-covalent binding. To verify that the SH– group of Cys476 is the reaction group, carbamidomethylation (Car) was performed to tag the substrate during the superhigh-resolution LC-MS/MS experiments. The DIII and HSA solutions were treated with Car to carbamidomethylate free SH– groups. The tagged sequences could be detected in the spectra (Supplementary Fig. 15). We observed that the Cys476 residue could not be labeled with Car as expected. It indicated that Cys476 is the binding site of Cl-containing dyes.

### Two independent stages are involved in the interaction between cyanine dyes and DIII

We next sought to understand the reaction mechanism between DIII and cyanine dyes and to confirm the increased brightness of the complex. We previously stated that the fluorescence intensities of almost all the cyanine and polymethine dyes increased when they were mixed with albumin or DIII; however, only Cl-containing dyes could form covalent bonds. These results indicated that the fluorescence enhancement could not be attributed to the formation of covalent bonds. We also stated that almost all the dyes exhibited high binding affinities toward albumin and DIII, indicating that the highly conjugated dyes can potentially bind to the hydrophobic pocket of albumin and its mutants through non-covalent supramolecular interactions. We therefore assume that cyanine dyes interact with DIII and its variants in two independent stages: In stage I, the dye inserts into the hydrophobic pocket of DIII and binds to the pocket through non-covalent interactions. At this stage, the brightness is enhanced though strengthening of TICT^16,40^. In stage II, a “clasp” is formed by the SH– group of Cys476 and the Cl–C group of the dye through nucleophilic substitution, which stabilizes Cl-containing dyes in the pocket (Figs. 1d and 3c). We believe that the side chains of the amino acid residues buried inside the pocket also play essential roles in the formation of the covalent bond.

### Binding to DIII improves the pharmacokinetics of Cl-containing cyanine dyes

**Fig. 4:**
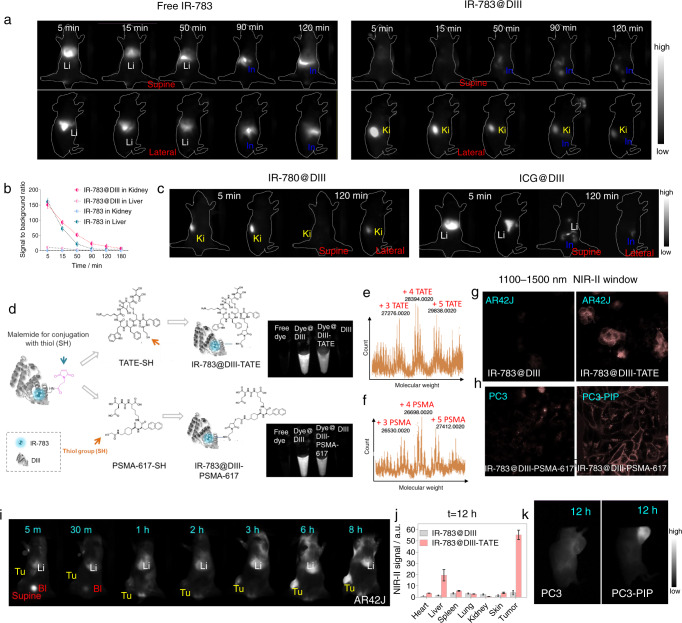
Dye@DIII exhibited improved pharmacokinetics compared with free dye and could be modified with targeting molecules. **a** NIR-II images of mice injected with free IR-783 or IR-783@DIII. Li: liver; Ki: kidney; In: intestine. **b** SBR of free IR-783 and IR-783@DIII in the liver and kidneys (*n* = 3). **c** NIR-II images of mice injected with IR-780@DIII or ICG@DIII. **d** Left: Schematic representation of the process for editing the DIII coating with the biofunctional molecules TATE and PSMA-617. Right: NIR-II images of free IR-783, IR-783@DIII, IR-783@DIII-molecular conjugate, and DIII. **e**, **f** UHPLC-MS analysis was used to confirm the successful modification of DIII with PSMA-617 (labeled as PSMA) or TATE. 3–5 molecules could be successfully conjugated to one DIII. **g**, **h** NIR-II microscopy images of IR-783@DIII-TATE uptake by SSTR-overexpressing AR42J cells (**g**) and IR-783@DIII-PSMA-617 uptake by PSMA-overexpressing PC3-PIP cells (**h**). **i**–**j** NIR-II images of an AR42J tumor-bearing mouse injected with IR-783@DIII-TATE (**i**) and the biodistributions of targeted and untargeted complexes at 12 h p.i. **j** (*n* = 3). a.u., arbitrary units. **k** NIR-II microscopy images of PC3 and PC3-PIP tumor-bearing mice injected with IR-783@DIII-PSMA-617. Data are presented as mean ± s.d. of three independent experiments. Source data are provided as a Source Data file.

To determine how the in vivo behavior of cyanine dyes is altered by a biocompatible coating of DIII, we performed NIR-II bioimaging of mice administered dye@DIII. When free IR-783 was administered to mice, the dye instantly accumulated in the liver (Fig. 4a, b). Soon after, the dye redistributed to the spleen and the intestinal tract, indicating rapid hepatobiliary clearance. In comparison, IR-783@DIII instantly accumulated in the kidneys and then rapidly redistributed to the bladder with no complex detected in the liver, indicating renal clearance (Fig. 4a, b). We also tested IR-780@DIII (a Cl-containing dye) and ICG@DIII (a Cl-free dye) to check the accuracy of our hypothesis that the pharmacokinetics of Cl-containing dyes and DIII complex could be altered (Fig. 4c, Supplementary Fig. 16). The behavior of IR-780@DIII was similar to that of IR-783@DIII, whereas ICG@DIII exhibited hepatobiliary clearance similar to that of free ICG, free IR-783 or free IR-780. Thus, DIII can function as a protective covalent coating for Cl-containing cyanine dyes and encompass them for protection, providing tailored pharmacokinetics for ultra-bright NIR-II imaging.

### Biofunctional modification of DIII coatings

As confirmed above, DIII increases the brightness of cyanine dyes and “packages” them for small molecule-like renal clearance. The DIII coating could also be modified with biofunctional molecules without altering the inserted dyes. For the reader’s convenience, the Cl-containing cyanine dye@protein would be abbreviated as “dye@protein” from this part, and the Cl-free dye would be clearly indicated. Consequently, dye@DIII composed of a biofunctionalized DIII coating could potentially function as ideal NIR-II molecular imaging probes. To this end, DIII was conjugated with the small targeting peptides octreotate (TATE), prostate-specific membrane antigen-617 (PSMA-617) and cyclic Arg-Gly-Asp (cRGD), which specifically bind to somatostatin receptor (SSTR), PSMA and integrin αVβ3, respectively. SH-modified TATE, PSMA-617 and cRGD were first reacted with maleimide-NHS ester. Following this, the purified products were conjugated to DIII (Fig. 4d). The reaction mixture consisted of DIII and small peptide at the ratio 1:5. Superhigh-resolution LC-MS/Matrix-assisted laser desorption ionization time-of-flight MS (MALDI-TOF MS) were used to confirm conjugation of IR-783@DIII with TATE, PSMA-617 and cRGD molecules (Fig. 4e, f, Supplementary Figs. 17–20). We first evaluated the fluorescence intensity of these complexes. The analysis exhibited that DIII modifications showed more or less decrease of brightness compared with that of dye@DIII (significant decrease was exhibited of dye@DIII-TATE and non-significant decrease was displayed of dye@DIII-PSMA and dye@DIII-cRGD, Fig. 4d, Supplementary Fig. 19). The decrease was considered to be impacted by the chemical reaction process, nonetheless, DIII modifications still showed ~1.4 times fluorescence enhancement over dye@HSA and tens of times over free dye integrated with decent targeting capacities, which still afforded satisfactory NIR-II bioimaging (Fig. 4g–k, Supplementary Figs. 19–20). Next, we used NIR-II microscopy to confirm the high targeting capacity of the peptide-modified fluorophores in AR42J (exhibit high expression of SSTR on the cell membrane), PC3-PIP (PSMA-transfected cells that exhibit high expression of PSMA on the cell membrane), and PC3 (PSMA-negative control) cells (Fig. 4g, h). Following these in vitro results, we investigated the targeting capacity of IR-783@DIII-TATE in AR42J tumor-bearing mice. Significantly large amount of the dye@DIII-TATE complex was found to accumulate in the tumor within 3 h post injection (p.i.) and increased with time (Fig. 4i, j). At 12 h p.i., the probe exhibited maximum tumor accumulation and the tumor-to-muscle fluorescence imaging ratio was as high as ~152 in 12 h (Fig. 4i). The biodistribution results indicated that only small amounts of the complex were detected in the liver and spleen at 12 h p.i. (Fig. 4j). We also prepared IR-783@HSA-TATE and IR-783@HSA-PSMA-617 using the same molar ratio and conjugation process as before for comparison. Each IR-783@HSA complex conjugated with comparable TATE or PSMA-617 molecules (verified by superhigh-resolution LC-MS in Supplementary Fig. 21). In vivo imaging of AR42J tumor-bearing mice injected with IR-783@HSA-TATE indicated that the probe accumulated in the liver at 5 min p.i. and then the intestines at 2 h p.i.. Only a small amount of probe accumulated in the tumor (Supplementary Fig. 22). After establishing the impressively targeted imaging capabilities of biofunctionalized dye@DIII, our next goal was to precisely tailor their pharmacokinetics for specific imaging purposes of different biological events.

### Genetic editing of DIII to produce a truncated subdomain for binding cyanine dyes

**Fig. 5:**
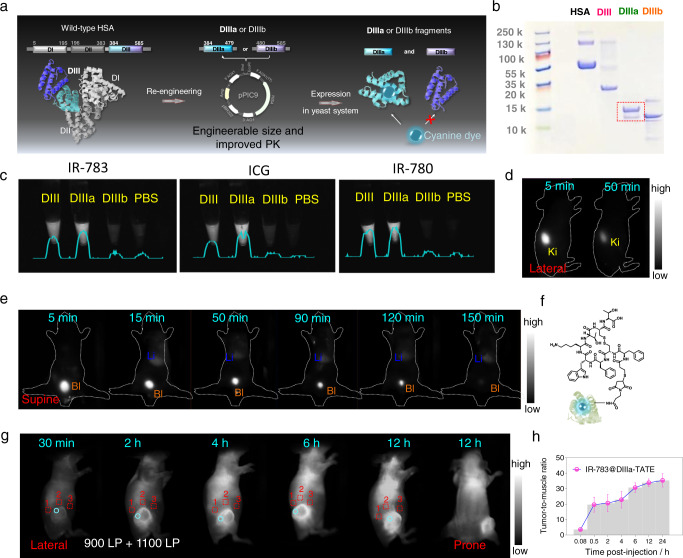
Dye@DIIIa exhibited improved pharmacokinetics compared with Dye@DIII and editable properties. **a** DIIIa was obtained using recombinant *Pichia pastoris* (pPIC9 strain). **b** Electrophoresis results for DIIIa and DIIIb. **c** NIR-II images of IR-783, ICG, and IR-780 complexed with DIII, DIIIa, or DIIIb. **d**–**e** NIR-II images of healthy mice injected with IR-783@DIIIa in lateral (**f**) and supine positions (**e**). Li Liver; Bl Bladder; Ki Kidneys. **f** Schematic figure of IR-783@DIIIa-TATE. **g**–**h** NIR-II images (**g**) and tumor-to-muscle ratio (**h**) of an AR42J tumor-bearing mouse injected with IR-783@DIIIa-TATE (*n* = 3). The red dotted squares were regions of interest (ROIs) of skin surrounding the tumor site, and the blue dotted circles were ROIs of tumor.

Analysis of the shotgun proteomics data revealed the presence of a minimal subunit (hydrophobic pocket) in DIII that binds cyanine dyes, resulting in increased brightness. Using this information, the independent DIIIa and DIIIb subunits were expressed by recombinant *Pichia pastoris* with their 3D structures retained (Fig. 5a)^41–43^. The samples were purified and SDS-PAGE confirmed the successful preparation of the DIII fragments (Fig. 5b). To identify which subunit is responsible for dye binding, we mixed IR-783 with DIIIa and DIIIb. The brightness of IR-783 increased severalfold when mixed with DIIIa but not DIIIb (Fig. 5c). The brightness of IR-783@DIIIa was comparable to that of IR-783@DIII. Further investigation revealed that DIIIa efficiently binds other dyes (ICG, IR-780; Fig. 5c). Thus, the genetically truncated DIII subunit containing the 3D structure responsible for binding cyanine dyes was identified to be DIIIa, and binding of dyes to this domain was sufficient to increase their overall brightness.

Since DIIIa is a fragment of DIII with approximately half its molecule weight, we hypothesized that the rate of renal excretion of IR-783@DIIIa would be higher than that of IR-783@DIII. The pharmacokinetic properties of IR-783@DIIIa were evaluated in healthy mice (Fig. 5d, e). As expected, the complex accumulated in the bladder faster than IR-783@DIII (Fig. 3a, supplementary Fig. 23). This result indicates that DIIIa could provide small molecule-like pharmacokinetic properties without compromising the brightness of dye@DIII, which could improve their overall safety by minimizing accumulation in major organs. We further conducted a pilot study in tumor-bearing mice to study the biological behavior and imaging abilities of peptide-conjugated IR-783@DIIIa (Fig. 5f). Two different conjugation methods (pre- and post-dye labeling) were used to determine if peptide conjugation influences the dye binding properties of DIIIa and the brightness of the resulting complexes (Supplementary Fig. 24a, b). When the pre-dye labeling method was used, brighter and more stable complexes were formed compared with the post-dye labeling method. The results revealed that peptide conjugation slightly reduced dye binding and decreased the brightness of the complexes (Supplementary Fig. 24c, d). We administrated pre-dye labeling IR-783@DIIIa-TATE to mice bearing AR42J tumor xenografts to investigate the targeting capabilities of the probe. Compared to IR-783@DIIIa, IR-783@DIIIa-TATE showed significantly higher accumulation in the tumor site (Fig. 5g, h).

### Albumin mimic containing three DIII domains (TDIII) increased brightness 2.7-fold

**Fig. 6:**
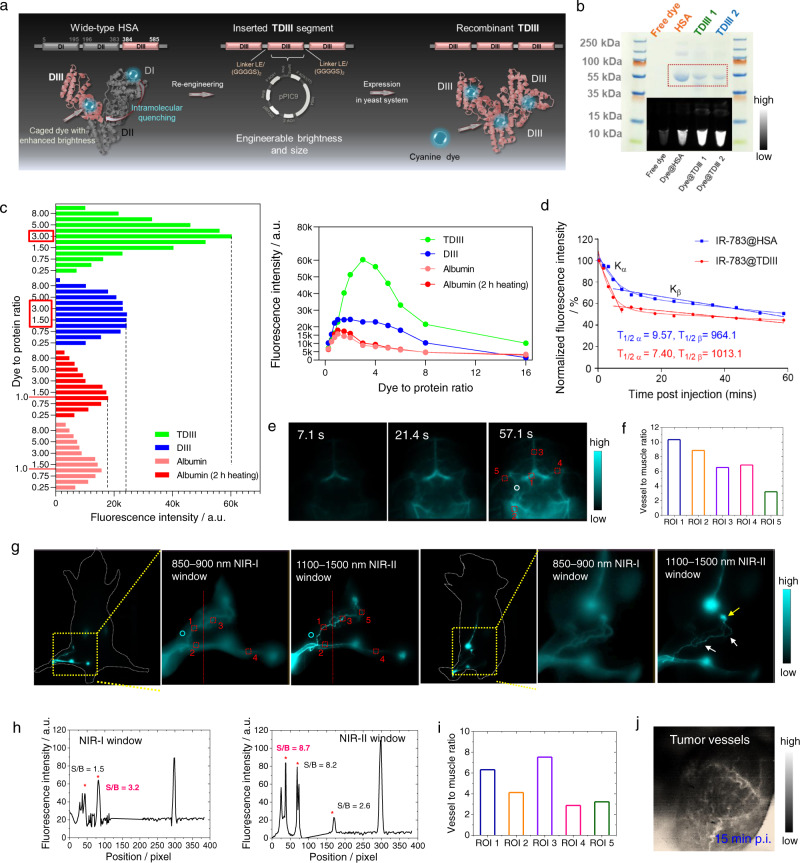
Dye@TDIII exhibited albumin-like pharmacokinetics and 2.7-fold brightness enhancement. **a** Schematic representation of TDIII expression using recombinant *Pichia pastoris* (pPIC9 strain). **b** Electrophoresis results of TDIII 1 (linkers: Leu-Glu) and TDIII 2 (linkers: -[Gly-Gly-Gly-Gly-Ser]_2_-). **c** The tendencies of mixtures of dye and albumin, DIII, TDIII as the dye-to-protein molar ratio increased from 0.25:1 to 16:1. **d** Comparison of half-life for IR-783@TDIII and IR-783@HSA using Non-linear regression and two-phase exponential decay analysis. **e**–**f** High-magnification (2.5×) NIR-II images (**e**) and ROI analysis (**f**) of blood vessels under the skull of a shaved mouse injected with IR-783@TDIII. **g**–**i** High-magnification (2.5×) NIR-I and NIR-II images (**g**), cross-sectional intensity profiles (**h**), and ROI analysis (**i**) of a mouse suffering from congenital lymphedema injected with IR-783@TDIII. The white arrows indicate the tortuous lymphatic vessels surrounding the inguinal LN and the yellow arrow indicates the edematous LN. **j** High-magnification (2.5×) NIR-II image of a mouse bearing a PC3 tumor at 15 min p.i. of IR-783@TDIII. Source data are provided as a Source Data file.

So far, we have shown that DIII can be modified with various targeting peptides/molecules to produce editable coatings for cyanine dyes. We have also shown that the coatings can be edited to form smaller complexes (dye@DIIIa) without altering the inserted cyanine dyes. However, the ideal pharmacokinetics of a fluorophore depend on the imaging application. For example, renally cleared probes are preferrable for molecular imaging due to their safety, while long-circulating probes are useful for vascular or lymphatic imaging. Long-circulating probes are also preferred for imaging-guided surgery. As dye@DIII/DIIIa are rapidly cleared through the renal system, they cannot function as long-circulating probes. IR-783@albumin was shown to be a long-circulating probe with a circulation time of 19 days, comparable to that of endogenous albumin^32^. The three domains of albumin (DI, DII, and DIII) are homologous (Supplementary Fig. 25), and the a and b subdomains on each domain have highly similar structures. The number of alpha helices and beta sheets in these subdomains have been shown to be the same and the disulfide bonds are formed at identical sites^44,45^. Therefore, we hypothesized that re-engineered proteins would retain some of the structural features and properties (such as long circulation time) of endogenous albumin if the DI, DII, or DIII units are substituted with each other. Hence, we re-engineered a recombinant HSA plasmid with 3 copies of the DIII sequence (triple DIII or TDIII) (Fig. 6a). To retain the natural properties of the fused protein and achieve albumin-like pharmacokinetic properties, we screened various natural linkers we typically used for high protein production and purification efficiency, *e.g*., leucine-glutamic acid (Leu-Glu) and (glycine-glycine-glycine-glycine-serine)_2_ (-[Gly-Gly-Gly-Gly-Ser]_2_-). After the recombinant *Pichia pastoris* clones were identified by polymerase chain reaction (PCR), TDIII was expressed in *Pichia pastoris* and verified by SDS-PAGE (Fig. 6b). And also, the hydrophobic sizes of these main agents were detected (Supplementary Fig. 26).

We performed a photometric titration experiment to evaluate the dye loading capacity of TDIII. DIII, HSA, BSA, and TDIII (1 µM, 100 µL) were mixed with various molar concentrations of IR-783 (*n* = 5). The dye-to-protein molar ratios investigated were 0.25:1, 0.5:1, 0.75:1, 1:1, 1.5:1, 2:1, 3:1, 4:1, 5:1, 6:1, 8:1 and 16:1. The samples were then exposed to a xenon lamp (780 nm) and the fluorescence intensity of each sample was detected by NIR-I (700–900 nm) and NIR-II (1100–1300 nm) detectors (Fig. 6c, Supplementary Fig. 27). For mixtures containing DIII, HSA, and BSA, the maximum fluorescence intensity was recorded when the molar ratio was 1:1, yet they showed distinct tendencies when the molar ratio increased (Fig. 6c, Supplementary Fig. 27). Consistent fluorescence intensities were recorded when the IR-783 to DIII molar ratios were 1:1, 1.5:1, 2:1, 3:1 and 4:1. In comparison, the fluorescence intensity for mixtures containing HSA and BSA decreased as the molar ratio increased. When the molar ratios were 0.5: 1, 0.75:1 and 1:1, the fluorescence intensities of the TDIII samples were comparable to those of the DIII samples. However, the fluorescence intensity for the 3:1 mixture of TDIII and dye was ~2.7-fold higher than that of the 1:1 mixture (Supplementary Fig. 27a). This result indicated that TDIII had approximately three times higher dye loading capacity than DIII; however, higher dye loading (>3:1), would also induce the self-quenching observed for HSA, BSA (>1:1). Meanwhile, the dye@albumin showed compared intensity tendency as that of the mixture with longer incubation at 60 °C (Fig. 6c). When dye@protein complex was heated, dye@DIII showed comparable fluorescence intensity at various temperatures, while dye@albumin and dye@TDIII showed fluorescence enhancement with the increase of temperature (<70 °C) (Supplementary Fig. 27b). The albumin variants are derived from natural serum albumin; thus, they were expected to show high biosafety. To study the biosafety of them, we evaluated the immunogenicity (Supplementary Figs. 28, 29) and performed hematology test of these variants-based complexes (Supplementary Fig. 30), and they showed high biosafety as expected.

### Use of TDIII for high-resolution blood vessel/lymphatic imaging and detection of congenital lymphedema

To determine if dye@TDIII has an extended circulation time, we performed in vivo NIR-II imaging of mice injected with IR-783@TDIII. To study the circulation time of IR-783@TDIII, we performed NIR-II dynamic imaging of healthy mice injected with IR-783@TDIII and IR-783@HSA (Supplementary Fig. 31). Since IR-783@TDIII showed severalfold brightness of IR-783@HSA, we normalized the fluorescence intensity to compare them. The half-life of IR-783@TDIII was similar to that of IR-783@HSA (Fig. 6d). The fluorescence signal at the heart was recorded for up to 3 h p.i. and pulsation of the heart was clearly visualized by dynamic imaging, and meanwhile, high-magnification (2.5×) NIR-II imaging of hindlimb vessels was recorded (Supplementary Movie 1). Next, we conducted real-time NIR-II imaging of vessels using IR-783@TDIII. Immediately after tail vein injection of the complex, video-rate imaging (76 frames per second) of blood flow in vessels of the hindlimb was performed at >1300 nm in the sub-NIR-II window (Supplementary Fig. 32). The migration speed of the complex in the vessel was calculated to be 4.06 ± 1.62 cm s^−1^. After successfully imaging superficial subcutaneous vessels, we aimed to delineate deep-seated vessels. High-magnification (2.5×) NIR-II imaging was performed in mice with shaved heads, and vessels underneath the skull and skin were imaged with high resolution (Fig. 6e, Supplementary Movie 2). Regions of interest (ROIs) were drawn on five selected vessels in the images. The signal-to-background ratio (SBR) values of the five ROIs ranged from 2.3 to 10.2 (Fig. 6f).

In addition, we performed real-time NIR-II imaging of popliteal, sciatic, and inguinal lymph nodes (LNs) and imaged lymphatic vessels. The ultra-bright IR-783@TDIII was able to illuminate the tiny lymphatic vessels with a resolution of 87.8 μm per pixel (Fig. 6g, h). NIR-II real-time imaging was used to monitor a mouse with congenital lymphedema containing tortuous and numerous lymphatic branches (Fig. 6g, Supplementary Movie 3), enabling successful delineation of the tortuous morphologies of the lymphatic vessels surrounding the inguinal LN. Comparison of the SBR values of selected ROIs in the NIR-I and NIR-II windows demonstrated the resolution advantage of NIR-II imaging (Fig. 6h, i). In parallel, high-magnification (2.5×) NIR-II imaging was also used to delineate PC3 tumor vessels (Fig. 6j).

### TDIII facilitated high-quality imaging-guided surgery

**Fig. 7:**
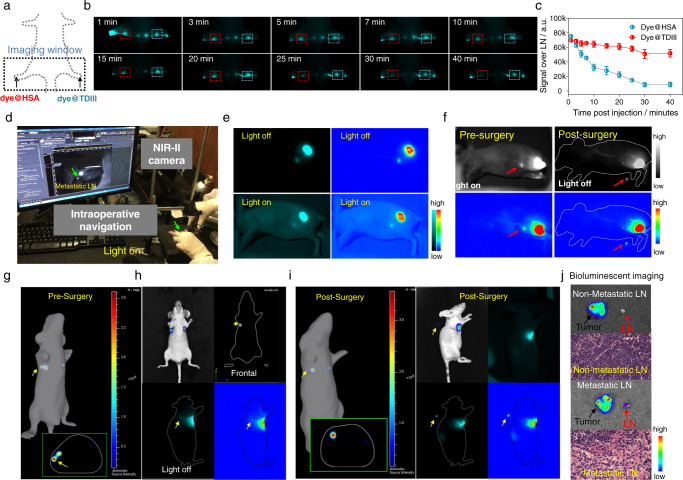
Dye@TDIII facilitated high-quality imaging-guided surgery. **a**–**c** In vivo photostability of IR-783@HSA and IR-783@TDIII during continuous laser exposure. **d** Intraoperative navigation platform for imaging-guided surgery in small animals (*n* = 3). **e**, **f** Intraoperative NIR-II images of mice intratumorally injected with IR-783@TDIII before and after resection of metastatic tumor-adjacent LNs in the presence and absence of room lighting. **g**–**j** Bioluminescence and NIR-II images of mice bearing orthotopic breast tumors with metastasis in the axillary LNs. Bioluminescence imaging was used to monitor tumor progression and metastasis to the sentinel LNs. The yellow arrows indicate the metastatic LNs. NIR-II imaging-guided resection of the metastatic LNs was performed following injection of IR-783@TDIII and confirmed by 2D and 3D bioluminescence imaging. Bioluminescence imaging and H&E staining confirmed that the excised LNs were metastatic (**j**). Source data are provided as a Source Data file.

Intraoperative navigation technique is widely used in the field of medicine. Currently, various cyanine dyes are used in the clinic, but these exhibit rapid photobleaching and clearance. We hypothesized that dye@TDIII could address the existing problems in the field. We first compared IR-783@TDIII to the clinically used ICG to study the optical properties of the probes (Supplementary Fig. 33). The same amount of IR-783@TDIII and ICG were injected bilaterally into the soles of the hind feet of a mouse, which were then exposed to a high-power laser and imaged. The brightness and photostability of ICG were significantly lower than those of IR-783@TDIII (Supplementary Fig. 33). Furthermore, IR-783@TDIII exhibited remarkably higher photostability compared with IR-783@HSA (Fig. 7a–c). This result indicated that IR-783@TDIII outperformed the clinically approved ICG and the previously reported IR-783@HSA^16^. Thus, dye@TDIII could be excellent candidates for imaging-guided surgery with translational potential.

We next performed imaging-guided surgery of sentinel LNs in a metastatic tumor model. Sentinel lymph node metastasis is the first step in metastasis and the dominant prognostic factor for staging many kinds of cancer, including melanoma, breast, cervical, uterine, and lung cancers. IR-783@TDIII was intratumorally injected into 4T1 tumor-bearing mice to pinpoint the location of the sentinel LNs. The sentinel LNs were identified 15 min after administration of the complex with high imaging contrast and photostability (Fig. 7d–f, Supplementary Fig. 34). Imaging-guided surgery was performed under bright room lighting with laser exposure. The sentinel LNs were efficiently pinpointed and precisely excised.

Next, we studied the imaging performance of the complex in an orthotopic 4T1 breast cancer model, which is known from animal and clinical studies to exhibit a high frequency of metastasis. Luciferase-transfected 4T1 tumor cells were inoculated into the mammary fat pads of mice and the mice were monitored until bioluminescence in the tumor and sentinel LNs was observed (Fig. 7g, h). Administration of IR-783@TDIII enabled capture of high-quality fluorescence images of the metastatic LNs under bright room lighting. Bioluminescence imaging and H&E staining confirmed metastasis in the sentinel LNs post LN removal (Fig. 7j).

## Discussion

The greatly attenuated tissue scattering and autofluorescence and consequent deeper tissue penetration in the NIR-II window compared with NIR-I opens many exciting avenues for deep and precise in vivo investigation of biological processes in clinical settings^46–49^. The clinically used cyanine dyes exhibit bright emission tails at wavelengths above 1000 nm^20^. This indicates that NIR-II imaging has reached a transformative stage with great potential for rapid clinical translation. However, existing NIR-II probes require complex chemical synthesis strategies to obtain tunable emission wavelengths, enhanced QYs, high biocompatibility, and satisfactory in vivo behavior. It remains challenging to generate NIR-II probes with precisely tailored pharmacokinetics and biofunctions using existing strategies.

The discovery of an albumin chaperone that functions as a brightness amplifier^16^ gave us an additional perspective on NIR-II probe synthesis: cyanine dyes as center chromophores could be packed inside biologically engineerable protein mutant shells to achieve enhanced brightness and satisfactory pharmacokinetics. In this study, we systematically engineered HSA in yeast and developed a series of albumin fragments and recombinant albumin mimics containing the minimal unit (binding pocket) for binding cyanine dyes. To our surprise, we observed that Cl-containing cyanine dyes formed covalent bonds with endogenous or mutant albumin, while Cl-free dyes formed non-covalent bonds. Some researchers have reported that Cys34 on albumin could covalently bind with IR-783^50,51^. However, the binding site was speculated on Cys34 since it is the only free cystine on albumin. In this study, we systematically studied and discovered a binding site (Cys476) on subdomain IIIa which could also covalently bond with Cl-containing cyanine dyes, and this finding has never been reported before. To confirm our finding, we used domain III or subdomain IIIa (without Cys34) instead of full albumin to react with Cl-containing dyes, and systematically studied the nucleophilic substitution of Cys476 and Cl-containing dyes. Based on this finding, we designed a series of recombinant albumin variants to chaperone Cl-containing dyes for in vivo NIR-II bioimaging. Furthermore, we determined that the interaction between cyanine dyes and albumin occurs in two independent stages and studied the mechanism of brightness enhancement. In the first stage, dyes bind to albumin or albumin mutants through non-covalent bonds, resulting in increased brightness though strengthened TICT. In the second stage, a “clasp” formed by the SH– group of Cys476 and the Cl–C group of Cl-containing dyes stabilizes the dyes through nucleophilic substitution. One group has observed some absorption spectra shifts (an immediate UV λ_max_ red-shift followed by a slow and gradual blue-shift after 90 min) but with no explanation^52^, while, according to this phenomenon, another group provided with a possible explanation that a noncovalent adduct would be slowly transformed covalent^51^. However, they did not provide with more evidence for the speculation.

This discovery provides a platform for the development of dyes that can attach themselves to serum albumin for improved pharmacokinetics. Cyanine dyes protected with a shell of albumin mutant or variant were shown to have enhanced optical properties. We also showed that the shells can be modified by fusion with a biofunctional peptide or conjugation with a small molecule. Additionally, the sizes of the bioengineered shells can be tuned to tailor the pharmacokinetic properties and clearance pathway. These excellent properties make the molecules suitable candidates for use in the fields of molecular imaging and imaging navigated surgery. In this study, we presented several applications in biofunctional imaging and imaging-guided surgery using complexes composed of bioengineered albumin fragments of various sizes with tailored pharmacokinetics and clearance pathways. We also designed an albumin mimic composed of three tandem DIII, which showed the highest brightness enhancement and generated dye complexes with albumin-like in vivo behavior.

In conclusion, we demonstrated an alternative strategy to conventional chemical modification techniques to develop targeted NIR-II probes. We developed a facile strategy to design bright and editable NIR-II fluorophores that exhibit tailored pharmacokinetic properties to address the problems of existing NIR-II probes. Our innovation uses existing cyanine dyes with peak emission at ~800 nm, which could achieve full color imaging in both the NIR-I and NIR-II windows to provide accurate diagnosis and guide surgery. We believe that development of cyanine dyes with peak emission at 1000–1500 nm will further promote our genetic engineering strategy. More importantly, this genetic engineering strategy would provide us with a platform for modifying NIR-II dyes and exhibit an additional perspective to design dyes. Our strategy can help the design and development of NIR-II probes with clinical translation potential in the near future.

## Methods

### Ethics statement

The investigators adhered fully to the “Guide for the Care and Use of Animals” by the NIH Clinical Center Animal Care and Use Committee (NIHACUC), and all animal work was conducted in compliance with protocols approved by the NIHACUC.

### Preparation of DIIIa and DIIIb

The aimed sequences of DIIIa and DIIIb were designed as follows. To make sure the connection part of DIIIa and DIIIb including, we designed an overlay segment with several amino acid residues (highlighted by blue color) on each of them.

The amino acid sequence of DIIIa (381–494):

LEKCCAAADPHECYAKVFDEFKPLVEEPQNLIKQNCELFEQLGEYKFQNALLVRYTKKVPQVSTPTLVEVSRNLGKVGSKCCKHPEAKRMPCAEDYLSVVLNQLCVLHEKTPVS

The amino acid sequence of DIIIb (490–585):

KTPVSDRVTKCCTESLVNRRPCFSALEVDETYVPKEFNAETFTFHADICTLSEKERQIKKQTALVELVKHKPKATKEQLKAVMDDFAAFVEKCCKA

To make sure the subdomains were successfully expressed and purified (since the small-molecule peptides or proteins are difficult to be expressed and purified using Pichia system), we used two strategies to achieve the small proteins: His tag and GST tag. Typically, a thrombin cleavage site (LVPRGS, green-colored sequence) was insert between tag and the targeting protein, respectively.

GST tag sequence:

MSPILGYWKIKGLVQPTRLLLEYLEEKYEEHLYERDEGDKWRNKKFELGLEFPNLPYYIDGDVKLTQSMAIIRYIADKHNMLGGCPKERAEISMLEGAVLDIRYGVSRIAYSKDFETLKVDFLSKLPEMLKMFEDRLCHKTYLNGDHVTHPDFMLYDALDVVLYMDPMCLDAFPKLVCFKKRIEAIPQIDKYLKSSKYIAWPLQGWQATFGGGDHPPKSDLEVLFQGPLGSPEFPGRLERPHRD

Hence, the GST-LVPRGS-DIIIa/DIIIb:

MSPILGYWKIKGLVQPTRLLLEYLEEKYEEHLYERDEGDKWRNKKFELGLEFPNLPYYIDGDVKLTQSMAIIRYIADKHNMLGGCPKERAEISMLEGAVLDIRYGVSRIAYSKDFETLKVDFLSKLPEMLKMFEDRLCHKTYLNGDHVTHPDFMLYDALDVVLYMDPMCLDAFPKLVCFKKRIEAIPQIDKYLKSSKYIAWPLQGWQATFGGGDHPPKSDLEVLFQGPLGSPEFPGRLERPHRDLVPRGSLEKCCAAADPHECYAKVFDEFKPLVEEPQNLIKQNCELFEQLGEYKFQNALLVRYTKKVPQVSTPTLVEVSRNLGKVGSKCCKHPEAKRMPCAEDYLSVVLNQLCVLHEKTPVS*

MSPILGYWKIKGLVQPTRLLLEYLEEKYEEHLYERDEGDKWRNKKFELGLEFPNLPYYIDGDVKLTQSMAIIRYIADKHNMLGGCPKERAEISMLEGAVLDIRYGVSRIAYSKDFETLKVDFLSKLPEMLKMFEDRLCHKTYLNGDHVTHPDFMLYDALDVVLYMDPMCLDAFPKLVCFKKRIEAIPQIDKYLKSSKYIAWPLQGWQATFGGGDHPPKSDLEVLFQGPLGSPEFPGRLERPHRDLVPRGSKTPVSDRVTKCCTESLVNRRPCFSALEVDETYVPKEFNAETFTFHADICTLSEKERQIKKQTALVELVKHKPKATKEQLKAVMDDFAAFVEKCCKA*

After achieved the above corresponding pPIC9K recombinant plasmid, we transformed them into GS115 strain. Specifically, the pPIC9K-DIIIa/DIIIb plasmid was linearized with XhoI and EcoR1, respectively. Then, GS115 strain was transformed by electroporation. Recombinant Pichia clones were isolated on RD plate. After the clones appeared in the plate, washed the clones with sterile water, and put them onto YPD + GS115 (1000 µg/ml) plate. Recombinant Pichia clones were identified by PCR.

PCR primes, suggest by Invitrogen yeast protocol, are documented as below:

5′AOX: 5′ ctgctgatagcctaacgttc 3′

3′AOX: 5′ gctgatcaggagcaagctcg 3′

Then, yeast (1 mL) was inoculated into 10 ml BMMD medium (2 % w / v glucose), and grown for 48 h on a rotary shaker of 250 r.p.m. at 30 °C. After that, 4 ml of each culture was inoculated in 2 × 200 ml BMMD media to grow for 120 h on a rotary shaker of 250 r.p.m at 30 °C. Proteins produced by recombinant Pichia were purified using the AlbuPure matrix (ProMetic BioSciences). Briefly, the supernatant was collected after centrifugation and filtered with 0.2 µm vacuum filter membranes (Millipore). Subsequently, a Pall Filtron LV system equipped with an Omega 10 kDa filter (LV Centramate cassette, Pall Filtron) was employed to further concentrate the filtered supernatant. The column was equilibrated with 50 mM sodium acetate pH 5.3 and loaded with the concentrated supernatant. After that, the column was washed with 10 column volume (CV) of equilibration buffer and 50 mM ammonium acetate pH 8.0 (10 CV) to elute the unbound fraction. The bound protein was eluted with a series of buffers, 50 mM ammonium acetate, 10 mM octanoate pH 8.0, 50 mM ammonium acetate, 30 mM sodium octanoate pH 8.0 or 200 mM potassium thiocyanate, respectively. Then, the eluted protein was concentrated and filtered against 10 CV of 50 mM NaCl by Vivaspin20 10 kDa PES (Sartorius). GP- HPLC was performed to quantify the HSA variants. Samples were chromatographed at a flow rate of 1 ml min^−1^ in 25 mM sodium phosphate, 100 mM sodium sulphate, 0.05 % (w / v) sodium azide, pH 7.0. The column was TSK G3000SWXL (Tosoh Bioscience), and the eluted proteins were detected by UV at 280 nm compared to an HSA standard. Finally, proteins were analyzed using SDS-PAGE in PhastGel^TM^ electrophoresis system and appropriate media.

### Preparation of TDIII

The DNA sequence of TDIII:

ATGAAGTGTTGTGCTGCTGCTGACCCACACGAATGTTACGCTAAGGTTTTCGACGAGTTCAAGCCATTGGTTGAGGAACCACAGAACCTGATCAAGCAGAACTGTGAGTTGTTCGAGCAGCTGGGTGAGTACAAGTTCCAGAACGCTTTGTTGGTCAGATACACCAAGAAGGTCCCACAGGTTTCCACTCCAACCTTGGTTGAAGTCTCCAGAAACCTTGGTAAGGTCGGTTCCAAGTGTTGTAAGCACCCTGAGGCTAAGAGAATGCCATGTGCTGAAGATTACTTGTCCGTCGTCTTGAACCAGTTGTGCGTCTTGCACGAAAAGACTCCAGTTTCCGACAGAGTTACCAAGTGCTGTACTGAGTCCTTGGTCAACAGACGTCCATGTTTCTCTGCTTTGGAGGTCGACGAAACCTACGTGCCAAAAGAGTTCAACGCTGAGACTTTCACTTTCCACGCTGACATCTGTACCCTGTCCGAAAAAGAGAGACAGATCAAGAAGCAGACTGCCTTGGTCGAGTTGGTTAAGCACAAGCCAAAGGCTACCAAAGAGCAGTTGAAGGCTGTTATGGATGACTTCGCTGCCTTCGTTGAGAAGTGTTGCAAGGCTTTGGAAAAGTGTTGCGCAGCTGCAGATCCTCATGAGTGTTACGCCAAAGTCTTTGATGAGTTTAAGCCCCTGGTCGAAGAACCCCAAAACTTGATTAAGCAAAACTGCGAACTGTTTGAGCAATTGGGCGAGTACAAATTTCAAAACGCCCTGCTGGTTAGGTACACTAAGAAAGTTCCTCAGGTGTCTACCCCAACTTTGGTCGAGGTTTCTAGGAACTTGGGTAAAGTGGGTTCTAAGTGCTGCAAACATCCAGAGGCCAAAAGAATGCCTTGCGCAGAGGACTACTTGTCTGTTGTTCTGAACCAGCTTTGTGTGCTGCACGAGAAAACCCCAGTCTCTGATAGAGTCACCAAATGTTGCACCGAGTCTCTGGTTAACCGTAGACCATGTTTTTCCGCCTTGGAAGTGGATGAGACTTACGTCCCTAAAGAGTTTAACGCCGAAACCTTTACCTTTCACGCCGATATCTGCACTTTGTCTGAGAAAGAGCGTCAGATTAAGAAACAAACCGCTCTGGTCGAACTTGTCAAGCACAAACCTAAAGCCACAAAAGAACAACTGAAGGCCGTCATGGACGATTTTGCCGCTTTTGTTGAGAAATGCTGTAAGGCCCTTGAAAAGTGCTGTGCCGCAGCCGATCCACATGAATGCTATGCTAAAGTGTTCGATGAGTTCAAACCACTTGTGGAAGAACCTCAGAATCTTATCAAACAAAATTGCGAGCTTTTCGAACAGTTGGGAGAGTATAAGTTTCAAAATGCCTTGTTGGTGCGTTACACAAAAAAGGTGCCTCAAGTCTCCACTCCTACTCTGGTTGAGGTTTCCCGTAACCTGGGAAAAGTTGGTAGCAAATGCTGCAAGCACCCCGAAGCTAAACGTATGCCTTGTGCCGAGGATTATCTGAGCGTTGTCTTGAATCAGCTGTGTGTCCTTCATGAGAAAACTCCCGTTTCTGACCGTGTCACTAAGTGTTGTACCGAAAGCTTGGTGAACAGAAGGCCTTGCTTTTCTGCTCTGGAAGTTGACGAGACATATGTTCCCAAAGAGTTCAACGCAGAAACATTCACATTTCATGCAGACATCTGCACACTTAGCGAGAAAGAAAGGCAAATCAAAAAGCAAACAGCCCTGGTTGAGCTGGTCAAACATAAGCCCAAGGCCACAAAAGAGCAGCTTAAAGCAGTAATGGACGATTTCGCTGCATTTGTCGAAAAGTGTTGTAAAGCCTAAGCGGCCGC

The amino acid sequence of TDIII (614AAs; 69.77 KDa; PI: 7.62)

MKCCAAADPHECYAKVFDEFKPLVEEPQNLIKQNCELFEQLGEYKFQNALLVRYTKKVPQVSTPTLVEVSRNLGKVGSKCCKHPEAKRMPCAEDYLSVVLNQLCVLHEKTPVSDRVTKCCTESLVNRRPCFSALEVDETYVPKEFNAETFTFHADICTLSEKERQIKKQTALVELVKHKPKATKEQLKAVMDDFAAFVEKCCKALEKCCAAADPHECYAKVFDEFKPLVEEPQNLIKQNCELFEQLGEYKFQNALLVRYTKKVPQVSTPTLVEVSRNLGKVGSKCCKHPEAKRMPCAEDYLSVVLNQLCVLHEKTPVSDRVTKCCTESLVNRRPCFSALEVDETYVPKEFNAETFTFHADICTLSEKERQIKKQTALVELVKHKPKATKEQLKAVMDDFAAFVEKCCKALEKCCAAADPHECYAKVFDEFKPLVEEPQNLIKQNCELFEQLGEYKFQNALLVRYTKKVPQVSTPTLVEVSRNLGKVGSKCCKHPEAKRMPCAEDYLSVVLNQLCVLHEKTPVSDRVTKCCTESLVNRRPCFSALEVDETYVPKEFNAETFTFHADICTLSEKERQIKKQTALVELVKHKPKATKEQLKAVMDDFAAFVEKCCKA*

The His tag was used in this sequence design since the TDIII is a big protein with molecule weight similarly equal to wild type albumin.

The amino acid sequence fused with cleavage site for thrombin and His tag (TDIII-LVPRGS-His tag).

MKCCAAADPHECYAKVFDEFKPLVEEPQNLIKQNCELFEQLGEYKFQNALLVRYTKKVPQVSTPTLVEVSRNLGKVGSKCCKHPEAKRMPCAEDYLSVVLNQLCVLHEKTPVSDRVTKCCTESLVNRRPCFSALEVDETYVPKEFNAETFTFHADICTLSEKERQIKKQTALVELVKHKPKATKEQLKAVMDDFAAFVEKCCKALEKCCAAADPHECYAKVFDEFKPLVEEPQNLIKQNCELFEQLGEYKFQNALLVRYTKKVPQVSTPTLVEVSRNLGKVGSKCCKHPEAKRMPCAEDYLSVVLNQLCVLHEKTPVSDRVTKCCTESLVNRRPCFSALEVDETYVPKEFNAETFTFHADICTLSEKERQIKKQTALVELVKHKPKATKEQLKAVMDDFAAFVEKCCKALEKCCAAADPHECYAKVFDEFKPLVEEPQNLIKQNCELFEQLGEYKFQNALLVRYTKKVPQVSTPTLVEVSRNLGKVGSKCCKHPEAKRMPCAEDYLSVVLNQLCVLHEKTPVSDRVTKCCTESLVNRRPCFSALEVDETYVPKEFNAETFTFHADICTLSEKERQIKKQTALVELVKHKPKATKEQLKAVMDDFAAFVEKCCKALVPRGSHHHHHH.

### Materials

Most of the cyanine dyes were purchased from Sigma-Aldrich. IR-12N3 was purchased from Nirmidas Biotech Co. Indocyanine Green (ICG, modified) for human injection was purchased from Dandong Yichuang Pharmaceutical Co., LTD. PBS was purchased from HyClone. Human Serum Albumin (HSA) and Bovine Serum Albumin (BSA) were purchased from Sigma-Aldrich. Fetal Bovine Serum (FBS) was purchased from Neuromics. Sucrose was purchased from Sigma-Aldrich.

### Kinetic binding assay

Binding affinity of cyanine dyes with albumin variants was determined by biolayer interferometry (BLI) by interaction using an Octet Red96 system (fortéBio), respectively. Because of the same protocol, binding affinity of IR-783 with HSA was taken as an example. IR-783 powder was freshly dissolved in anhydrous DMSO (26.7 mM). Then, IR-783 is serially diluted into different concentrations (100, 50, 25, 12.5, 6.25, 3.125, 1.56 μM) by PBS. The assay protocol was briefly described as follows. After washing with 1 × PBS for 60 s, biotinylated HSA (1 μg/mL) was loaded to the biosensor for 600 s, and after another 60 s washing, quenching with biocytin was performed for 180 s. Followed by another 60 s washing, association for 600 s and dissociation for another 600 s were performed in turn. Data was calculated and analyzed using Octet Analysis software v 7.0.

### Synthesis of Dye@HSA (HSA variants) complex

Synthesis of dye@HSA and dye@HSA-domains/variants complex shared the same protocol and IR-783-DIII complex was taken as an example. DIII powder was dissolved in 1XPBS with a concentration of 20 mg/mL (855 µM); IR-783 powder was freshly dissolved in anhydrous DMSO (26.7 mM). 500 µL DIII PBS solution was first added in 500 µL PBS under slight vortexing. Then, 8, 16, 32, 48, 96 μL of the IR-783 (26.7 mM) was added into the DIII solutions, respectively (molar ratios were 0.5:1, 1:1, 1:2, 1:3, 1:4). Finally, the suspension was vortexed for 30 s and heated at 60 °C for 10 min.

### Dye@DIII/DIIIa/TDIII-TATE/PSMA-617

Since we do not know if the conjugation of TATE/PSMA-617 would impact the binding of IR-783, we attempted to add IR-783 before or after the conjugation of TATE/PSMA. Results indicated that both routes did not lead to obvious fluorescence diminishing; hence, we conclude that tailoring DIII does not impact the in vitro and in vivo behavior of the fluorophores. IR-783@DIII or DIIIa (TDIII) was reacted with maleimide-PEG-NHS ester (Sigma) at RT. for 4 h under shaker. Then products (IR-783@DIII-maleimide, IR-783@DIIIa-maleimide, IR-783@TDIII-maleimide) were filtered with 10 k filter against PBS for 5 times to move unreacted maleimide-PEG-NHS ester. The maleimide-PEG-NHS labeled compounds were incubated with PSMA-SH and TATE-SH peptides (molar ratio of protein and peptide was 1:5), as described in our previous paper. After that, the conjugates were washed with 10 K filter against PBS for 5 times.

### Characterization

UV-Vis-NIR spectrophotometer (Cary 6000i) with background correction was employed to measure the optical absorption spectrum. Fluorescence spectrophotometry was carried out on a Hitachi F-7000 fluorescence spectrophotometer. Fluorescence microscopy was performed on an Olympus fluorescence microscope.

### Matrix-assisted laser desorption ionization time-of-flight mass spectrosocopy (MALDI-TOF MS)

DIII-cRGD was mixed with HCCA (α-cyano-4-hydroxycinnamic acid) in a 1:1 ratio, 2 μL of sample and 2 μL of HCCA. Sample matrix was mixed and loaded onto a MALDI target plate (MTP) 384 by direct droplet method. It was allowed to dry for a few minutes. MALDI-TOF mass spectrometry was performed on Autoflex maX (Bruker) instrument. Finally, the data was plotted using Graphpad 8.0.

### NIR-II imaging

Mice were shaved using Nair hair removal cream and anesthetized using isoflurane before placing them for injection of imaging agents. For each imaging experiment, at least 3 mice were used as a parallel group cohort. All NIR-II images were collected on a Princeton InGaAs array. The excitation laser was an 808 nm laser set-up. Emission was typically collected with different long pass filters. A lens set was used for obtaining tunable magnifications, ranging from 1× (whole body) to 2.5× (high magnification) by changing the relative position of two NIR achromats (200 mm and 75 mm, Thorlabs). NIR-II microscopy was built based on the previous report, and the excitation laser was a 785 nm laser set-up.

### Cell lines and cell culture

AR42J (from ATCC) were cultured in F-12k medium with 20% heat-inactivated FBS and 1% penicillin and streptomycin. PC3 (from ATCC), PC3-PIP (from NIBIB at NIH) and 4T1-fluc cell lines (from ATCC) were cultured in RPMI-1640 medium with 10% heat-inactivated FBS and 1% penicillin and streptomycin. Cells were grown in a humidified atmosphere (5% CO_2_, 37 °C). All cells were tested to be free of mycoplasma.

### Animal studies

Nude mice (female, 6-8 weeks), C57BL/6j mice (female, 6–8 weeks) and balb/c mice (female, 6–8 weeks) were purchased from Jackson’s Laboratory (Bar Harbor, ME). Bedding, nesting material, food, and water were provided ad libitum. Ambient temperature was controlled at 20 to 22 °C with 12 h light/12 h dark cycles. AR42J or PC3 cells (5.0 × 10^6^) were inoculated to nude mice to grow subcutaneous tumor, respectively. 4T1-fluc cells (1.0 × 10^5^) were inoculated in the mammary fat pad to establish the orthotopic mammary tumor model/metastatic tumor model.

### Flow cytometry

Female C57BL/6 mice (6–8 weeks) were intravenously injected with PBS, DIII, IR-783@DIII, IR-783@HSA, IR-783@DIIIa, and IR-783@TDIII, respectively. Peripheral blood was collected to analyze subtypes of lymphatic cells. Briefly, blood was collected from the treated mice on day 3, day 7 and day 14. Blood cells were enriched by centrifugation. Red blood cells were lysed using ACK lysis buffer for 10 min at room temperature. Then, cells were washed twice in PBS and stained with the according antibodies for 15 min. After that, cells were washed with FCS buffer (PBS buffer with 0.1% FBS), and resuspended for flow cytometric analysis. Flow cytometry was conducted on a Beckman CytoFlex S flow cytometer. Data were analyzed using FlowJo V10 version.

### Quantum chemical calculations

The ground state (S0) geometries of IR-783 and ICG molecules were optimized using the B3LYP-GD3GJ/6-31 G(d) method dispersion correction. The excited state (S1) properties were calculated using the time-dependent (TD) LC-BLYP*/6-31 G(d) method with the polarizable continuum model (PCM). The HOMO and LUMO distributions were displayed using VMD code. All the (TD)DFT calculations were performed using Gaussian 16 software.

### Reporting summary

Further information on research design is available in the Nature Research Reporting Summary linked to this article.

## Supplementary information

Supplementary Information

Reporting Summary

Description of Additional Supplementary Files

Suppl Movie 1

Suppl Movie 2

Suppl Movie 3

## Data Availability

The authors declare that all the data related with this study are available within the paper, supplementary file, and source data. Source data are provided with this paper.

## Source data

Source Data
