# Supplementary material for: A genetic engineering strategy for editing near-infrared-II fluorophores
Source: Nat Commun. 2022 May 23;13:2853. doi: 10.1038/s41467-022-30304-9 (PMC9127093; doi:10.1038/s41467-022-30304-9)
Supplement: Supplementary file 1 — Supplementary Information [file 41467_2022_30304_MOESM1_ESM.pdf]

Supplementary Information

A genetic engineering strategy for editing near-infrared-II fluorophores

Tian et al.

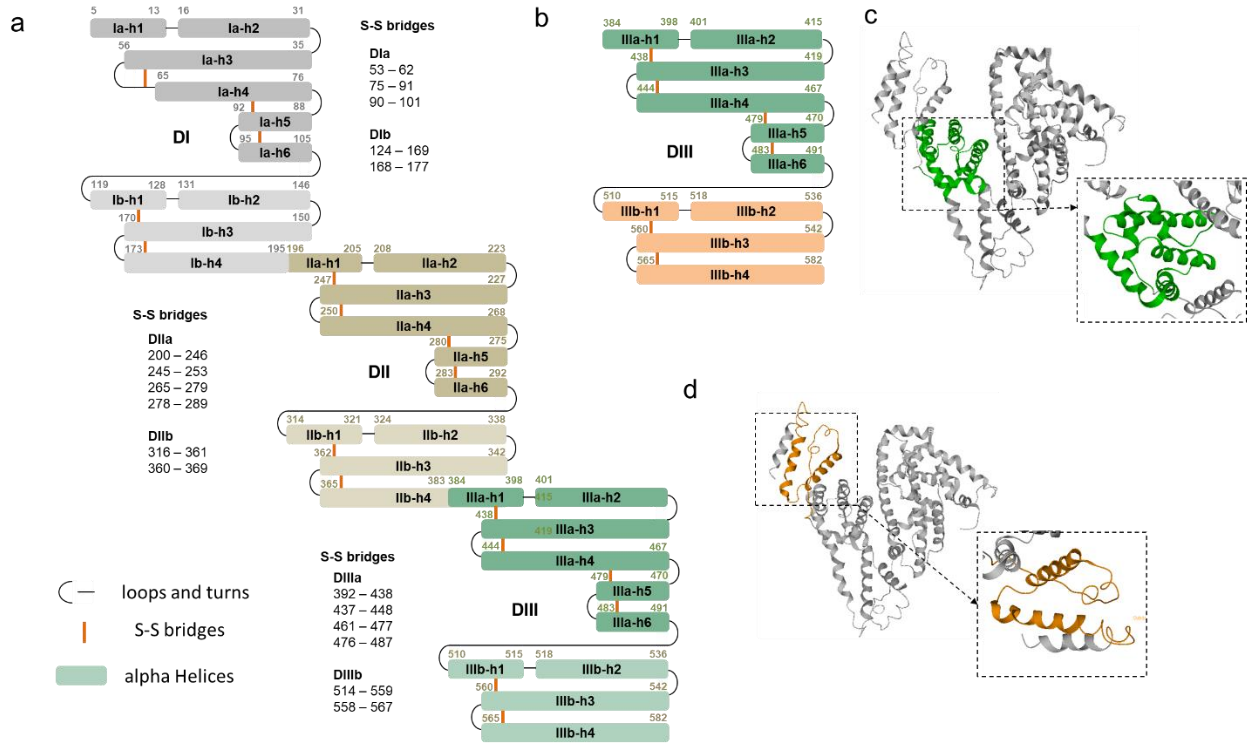

Supplementary Fig. 1. **Schematic structure of HSA and HSA sub-domains.** a) Schematic diagram of domains and subdomains of HSA. The secondary structure elements and disulfide bridges of HSA were exhibited on each domain. Helices are represented by rectangles with loops and turns by thin lines. Disulfide bridges are drawn in thick orangelines. b) Domian III is composed of Domain IIIa (DIIIa, green color) and Domain IIIb (DIIIb, orange color) in series. The spatial sites of c) DIIIa and d) DIIIb are highlighted in green/orange color in the cystal struture of serum albumin (PBD: 1E78).

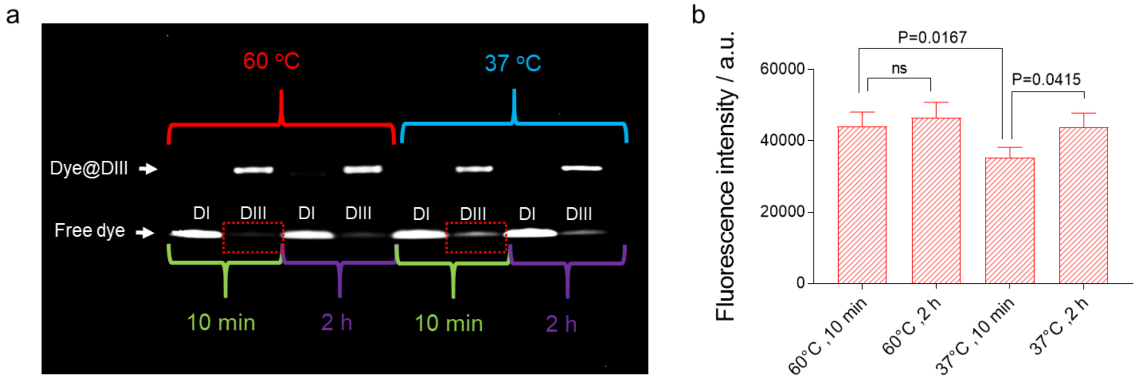

Supplementary Fig. 2. Heating mixture of IR-783 and DIII increased the binding and fluorescent efficiency. a) Electrophoresis analysis of mixtures of IR-783 with DI and DIII (10 min or 2 h post-heating) under NIR-II fluorescence imaging. b) Quantification of fluorescence intensity of mixtures with different incubation time at different temperatures, respectively ($n = 3$). Data are presented as mean ± s.d. of 3 independent experiments. ns: not significant ($p > 0.05$), unpaired student T-test, two-sided.

Supplementary Table 1. Comprehensive study on optical properities between cyanine dyes and dye@albumin/its variants.

	Absorption peak / nm	Emission peak / nm [a]	Extinction coefficient / $M^{-1}cm^{-1}$	NIR-II quantum yields / % [b]	NIR-II quantum efficiency [c]
**IR-783**	775 nm	810 nm	$0.95x10^5$	Not detectable	-
**IR-783@BSA**	794 nm	810 nm	$1.82x10^5$	0.85% to 8.50%	0.155 to $1.55*10^6$
**IR-783@HSA**	796 nm	809 nm	$1.95x10^5$	0.91% to 9.08%	0.177 to $1.77*10^6$
**IR-783@DIII**	790 nm	810 nm	$2.51x10^5$	0.97% to 9.73%	0.244 to $2.44*10^6$

a) Emission spectra were recorded by 808 nm excitation.

b) Reference: QY of Hipco SWCNT is 0.04% to 0.4%.

c) Quantum efficiency = Extinction coefficient * quantum yield.

d) Due to differences in batch-to-batch of original SWCNTs and production processes, the QY of our freshly made HiPCO SWCNTs always has variation. We presumed the previous QY value of IR-783@BSA was overestimated due to the property-discrepancy of SWCNTs reference (Sci. Adv. 2019, 5, eaaw0672).

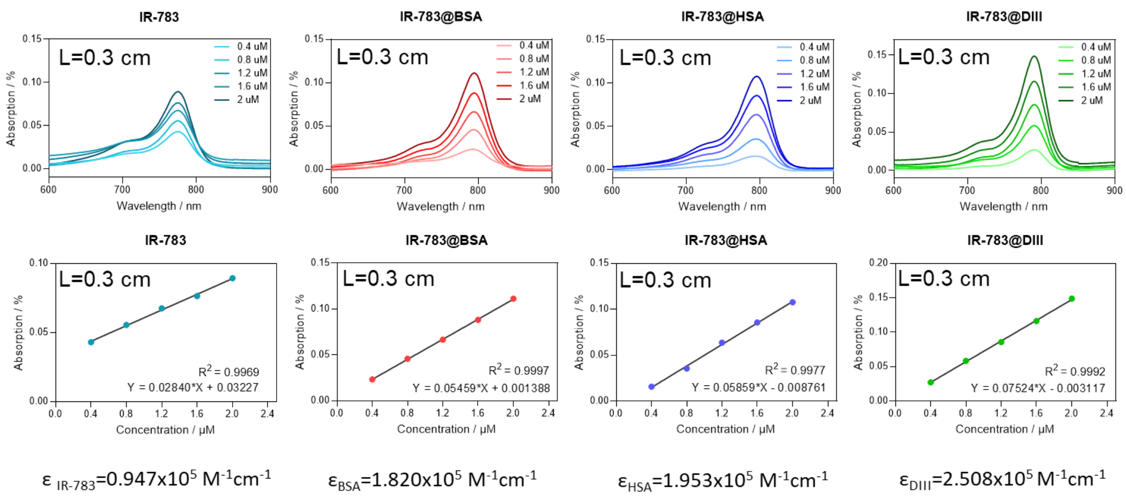

Supplementary Fig. 3. Molar absorption coefficient of IR-783@albumin/its variants.

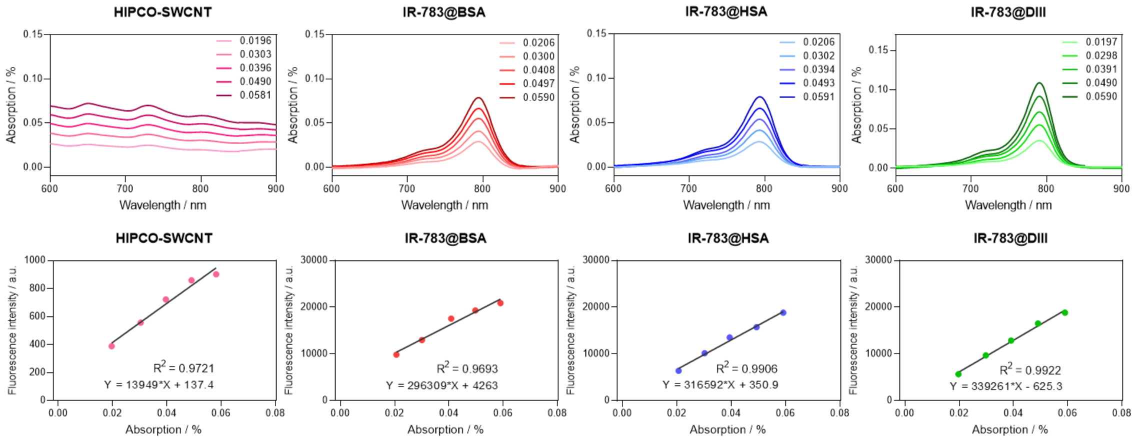

Supplementary Fig. 4. NIR-II quantum yield of IR-783@albumin/its variants.

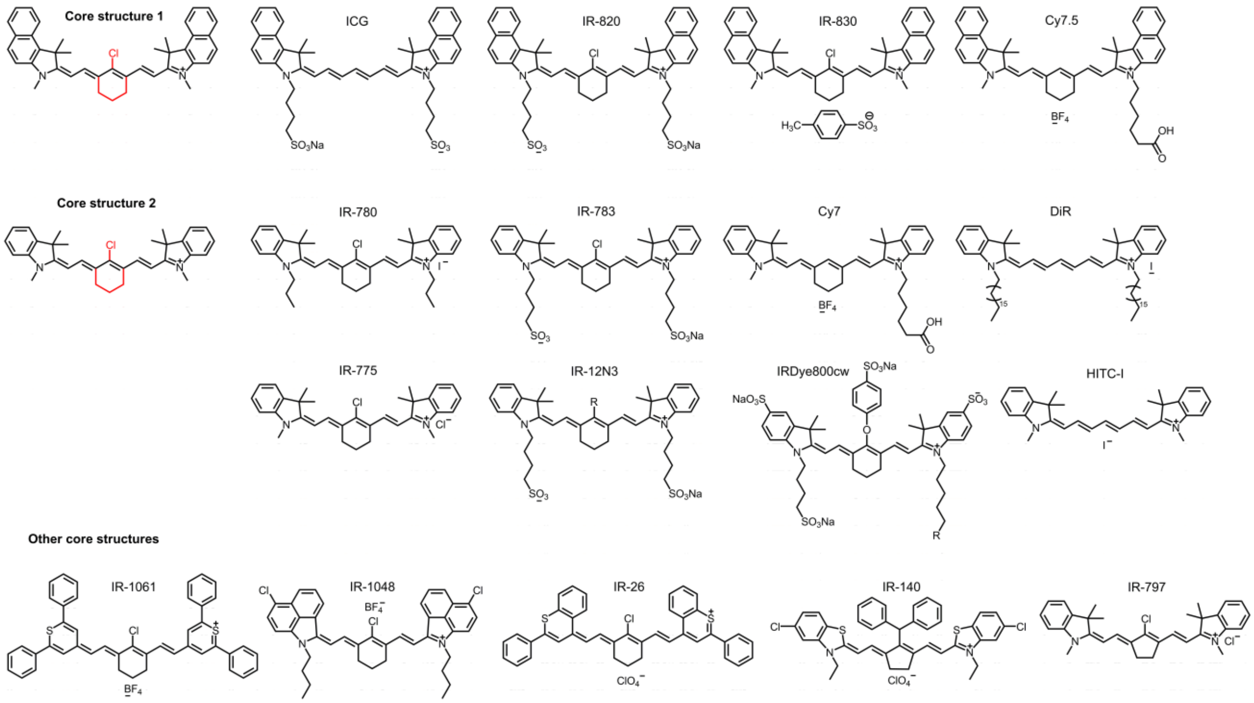

Supplementary Fig. 5. **Shared core and molecular strutures of cyanine dyes/polymethine dyes in current study**. The precise interaction between dyes and albumin (sub-domains) was systematically investigated. This interaction was experimentally determined to contain supramolecular interaction for most of attempted dye structures and covalent bonding for chloro-containing cyanine dyes with two exact binding sites on the albumin (sub-domains).

Supplementary Table 2. K_d, K_{on} and K_{off} for IR-783, IR-780 and ICG with DIII.

**Name (with DIII)**	**Goodness of fit (R^2)**	**K_{on} (1/M ▪s)**	**K_{off} (1/s)**	**K_d (nM)**
IR-783	0.9090	4.094×10^5	0.1001	0.57
IR-780	0.9923	$3.\ 002\times10^5$	0.1247	0.93
ICG	0.9617	1.872×10^5	0.3029	1.8

Note: K_d is the equilibrium dissociation constant, a ratio of k_{off} / k_{on}, between the dye and albumin/its variant. nM: nanomolar (Molar concentration). k_{off} : Association constant; k_{off} : Dissociation constant; nM: nanomolar (Molar concentration).

Supplementary Table 3. K_d, K_{on} and K_{off} for IR-783, IR-780 and ICG with HSA

Name (with HSA)	Goodness of fit (R^2)	K_{on} (1/M ▪s)	K_{off} (1/s)	K_d (nM)
IR-783	0.9902	$2.\ 512\times10^5$	0.2038	1.7
IR-780	0.9894	1.345×10^5	0.3947	1.9
ICG	0.9935	4.713×10^5	0.8200	1.5

Note: Note: The meaning of K_d ,k_{off} and k_{on} are presented footnote of table.

Supplementary Table 4. Comprehensive studing on binding affinities between cyanine/polymethine dyes and HSA through Bio-Layer Interferometry (BLI).

Name	K_d (nM)	
	**With DIII**	**With HSA**
ICG	1.8	1.5
IR-783	0.57	1.7
IR-780	0.93	1.9
IR-820	2.3	3.6
IR-830	0.99	3.2
IRdye800cw	65.3	174.0
cy7	13.2	4.1
cy7.5	20.6	12.2
IR-140	10.2	12.3
IR-1048	2.7	21.2
IR-1061	3.1×10^2	7.7×10^2
IR-12N3	0.97	4.7
DiR	2.70×10^5	1.44×10^5
HITC	1.03×10^2	1.97×10^2
IR-775	2.91	11.1
IR-797	12.2	3.97
IR-26	6.02×10^2	9.9×10^2

Note: The meaning of K_d ,k_{off} and k_{on} are presented footnote of table 2.

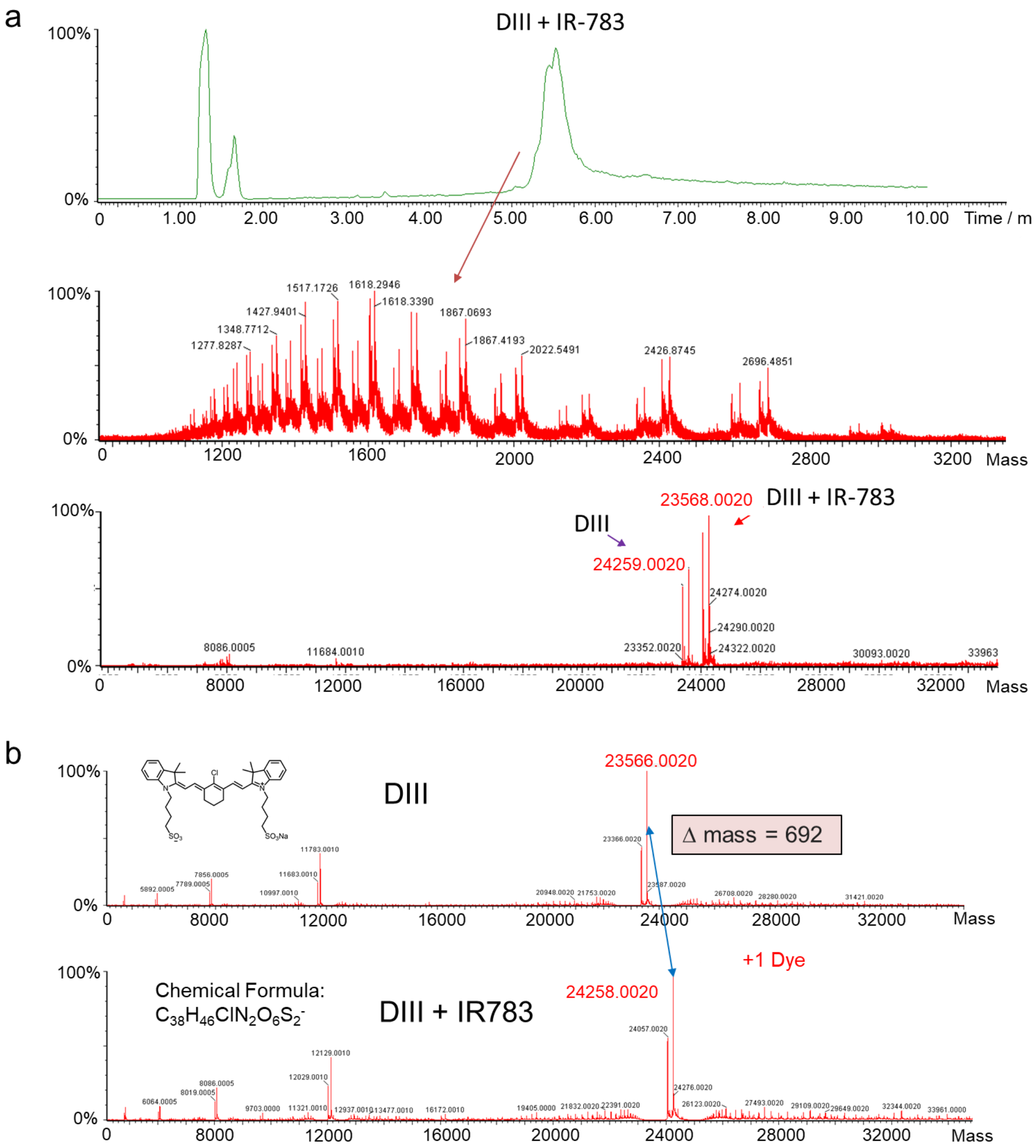

Supplementary Fig. 6. Superhigh resolution LC/MS confirmed that one DIII molecule could bind with one and only one IR-783. a) Superhigh resolution LC/MS survey of DIII mixed with IR-783. b) Superhigh resolution LC/MS survey of free DIII compared with IR-783@DIII complex. The chemical formula of IR-783 is $C_{38}H_{46}ClN_2NaO_6S_2$ with exact mass 748.24. Note: IR-783 was freely mixed with DIII at room tempreture, and then the mixure was analysized.

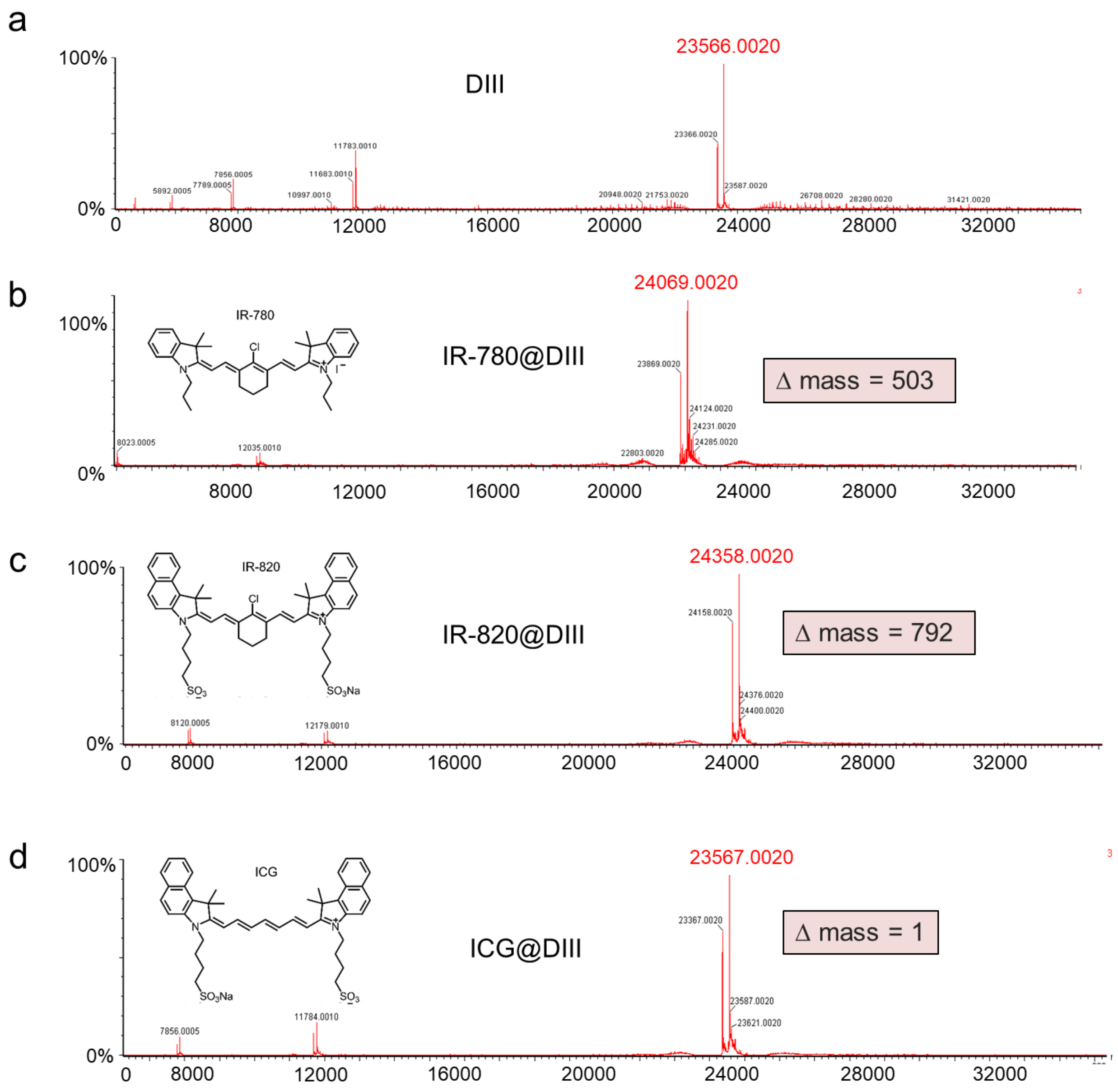

Supplementary Fig. 7. The molecular weight of DIII and dyes@DIII complex was confirmed by superhigh resolution LC/MS. The exact mass survey of DIII (a), IR-780@DIII (b), IR820@DIII (c), ICG@DIII (d) revealed the detailed binding ways of DIII and different cyanine dyes.

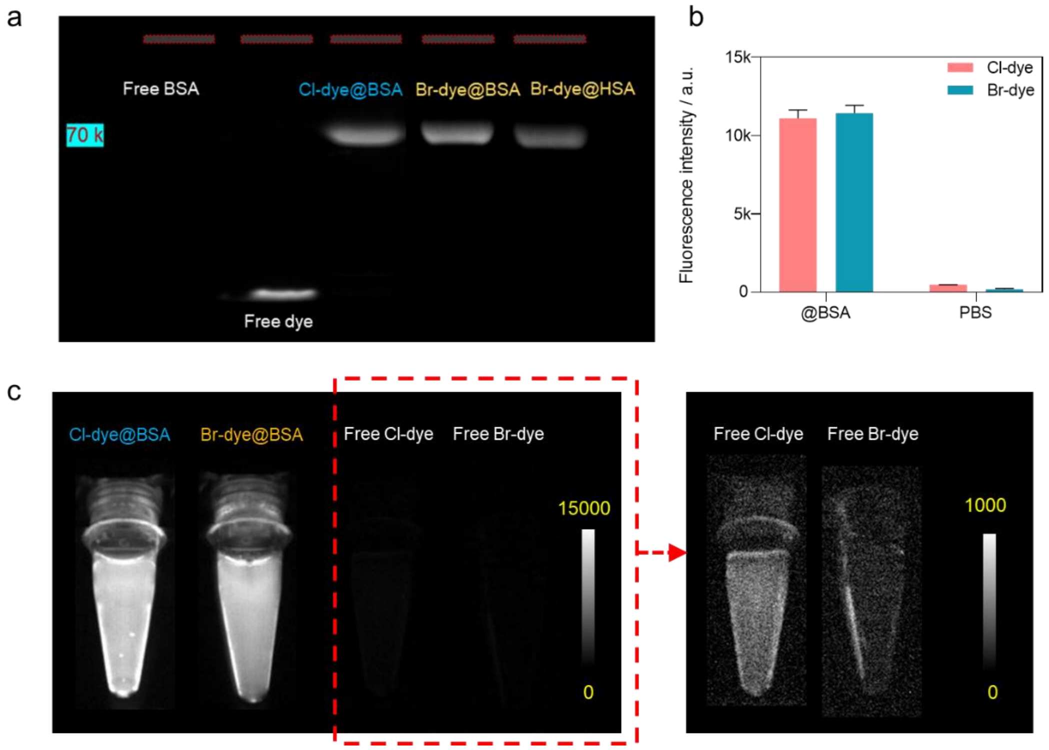

Supplementary Fig. 8. Br-dye was synthesized and its albumin complex was evaluated with NIR-II imaging. Comparison of binding a) and brightness b-c) of Cl-dye and Br-dye mixed with albumin, respectively (n = 3).

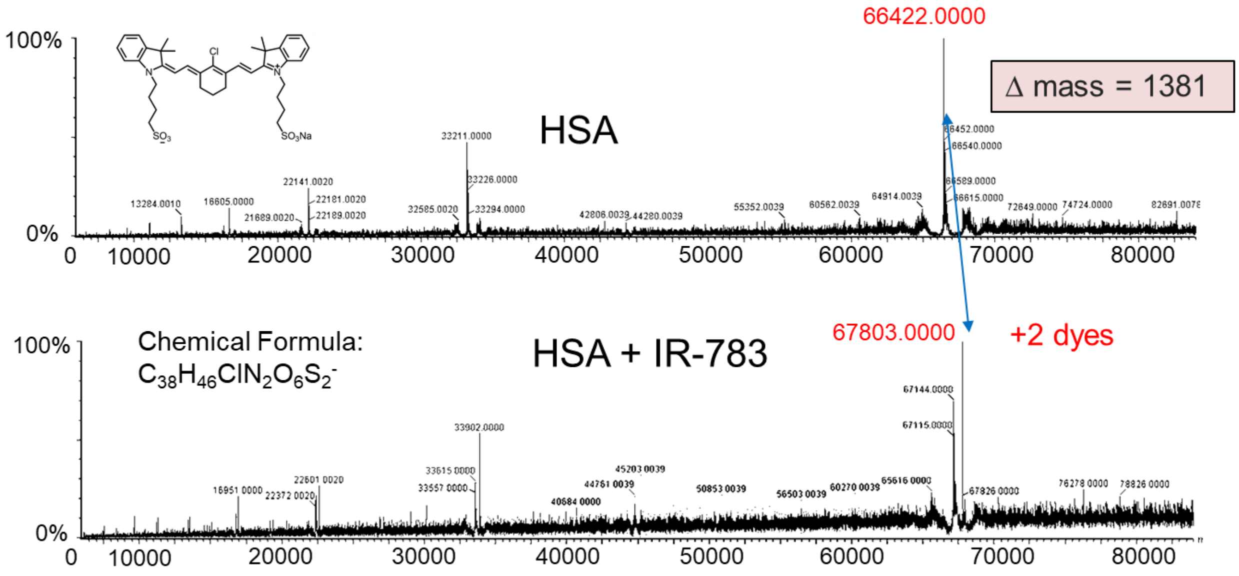

Supplementary Fig. 9. The exact mass survey of HSA and IR-783@HSA complex. It showed that one HSA molecule could bind with two cyanine dyes in the case of IR-783 mixed with HSA.

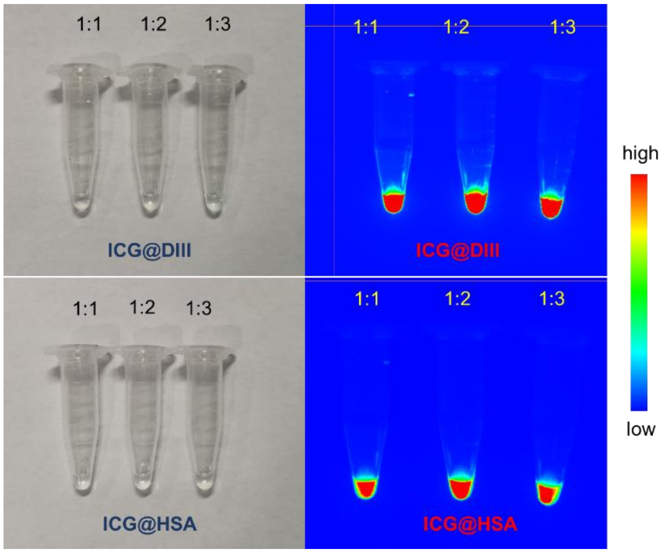

Supplementary Fig. 10. NIR-II images of ICG@DIII and ICG@HSA with various of protein-to-dye molar ratios. It showed the fluorescence intensities of both of ICG@HSA and ICG@DIII complex were stable when the ICG loading was increasing.

a Workflow for the metaproteomic analysis of binding site(s) confirming

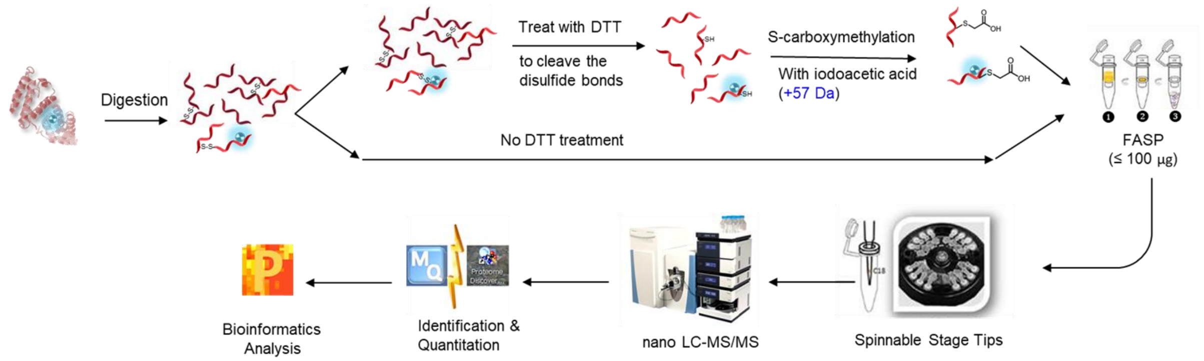

b Digestion enzymes and cleavage sites on DIII

Enzyme									
Trypsin	1	KQNCELFEQL	GEYKFQNALL	VRYTKKVPQV	STPTLVEVSR	NLGKVGSKCC	KHPEAKRMPC	AEDYLSVVLN	QLCVLHEKTP
	81	VSDRVTKCCT	ESLVNRRPCF	SALEVDETYV	PKEFNAETFT	FHADICTLSE	KERQIKKQTA	LVELVKHKPK	ATKEQLKAVM
	161	DDFAAFVEKC	CKADDKETCF	AEEGKKLVAA	SQAALGL				
Asp-N	1	KQNCELFEQL	GEYKFQNALL	VRYTKKVPQV	STPTLVEVSR	NLGKVGSKCC	KHPEAKRMPC	AEDYLSVVLN	QLCVLHEKTP
	81	VSDRVTKCCT	ESLVNRRPCF	SALEVDETYV	PKEFNAETFT	FHADICTLSE	KERQIKKQTA	LVELVKHKPK	ATKEQLKAVM
	161	DDFAAFVEKC	CKADDKETCF	AEEGKKLVAA	SQAALGL				
Glu-C	1	KQNCELFEQL	GEYKFQNALL	VRYTKKVPQV	STPTLVEVSR	NLGKVGSKCC	KHPEAKRMPC	AEDYLSVVLN	QLCVLHEKTP
	81	VSDRVTKCCT	ESLVNRRPCF	SALEVDETYV	PKEFNAETFT	FHADICTLSE	KERQIKKQTA	LVELVKHKPK	ATKEQLKAVM
	161	DDFAAFVEKC	CKADDKETCF	AEEGKKLVAA	SQAALGL				
Lys-C	1	KQNCELFEQL	GEYKFQNALL	VRYTKKVPQV	STPTLVEVSR	NLGKVGSKCC	KHPEAKRMPC	AEDYLSVVLN	QLCVLHEKTP
	81	VSDRVTKCCT	ESLVNRRPCF	SALEVDETYV	PKEFNAETFT	FHADICTLSE	KERQIKKQTA	LVELVKHKPK	ATKEQLKAVM
	161	DDFAAFVEKC	CKADDKETCF	AEEGKKLVAA	SQAALGL				

Supplementary Fig. 11. An unbiased shotgun proteomics approach was performed to identify the nucleophilic substitution reaction site between Cl-C group of cyanine dyes and protein residue(s). a) The workflow for the metaproteomic analysis of binding site(s) confirmation. b) Digestion enzymes and their targeting cleavage sites on DIII.

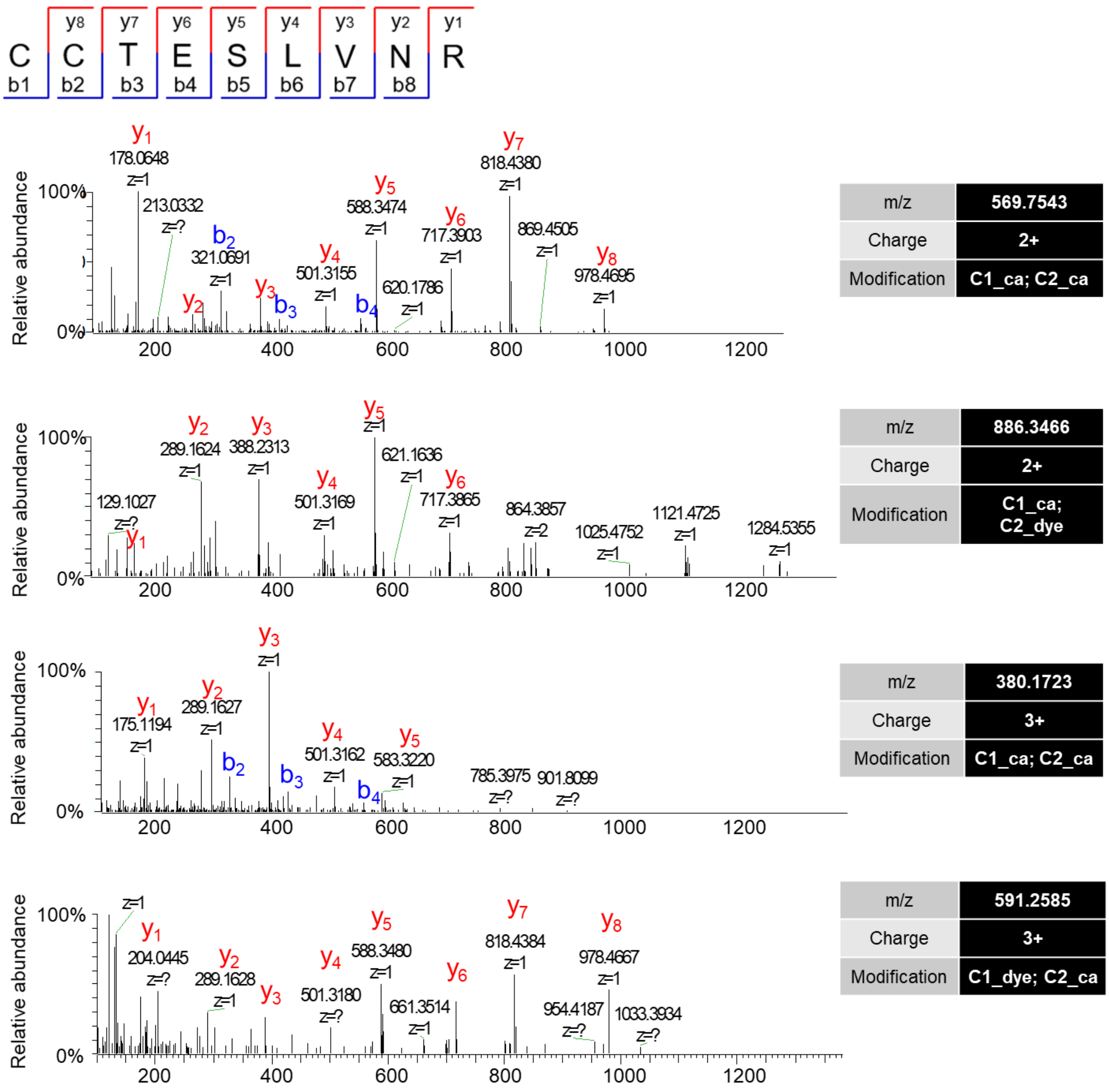

Supplementary Fig. 12. The sequence of interest (TESLVNR) was confirmed by above-mentioned shotgun proteomics approach. The exact binding residue on this sequence, the b and y ion series were analyzed in detail. Results verified that Cys 476 was the binding site for nucleophilic substitution of Cl-C group on cyanine dye backbone.

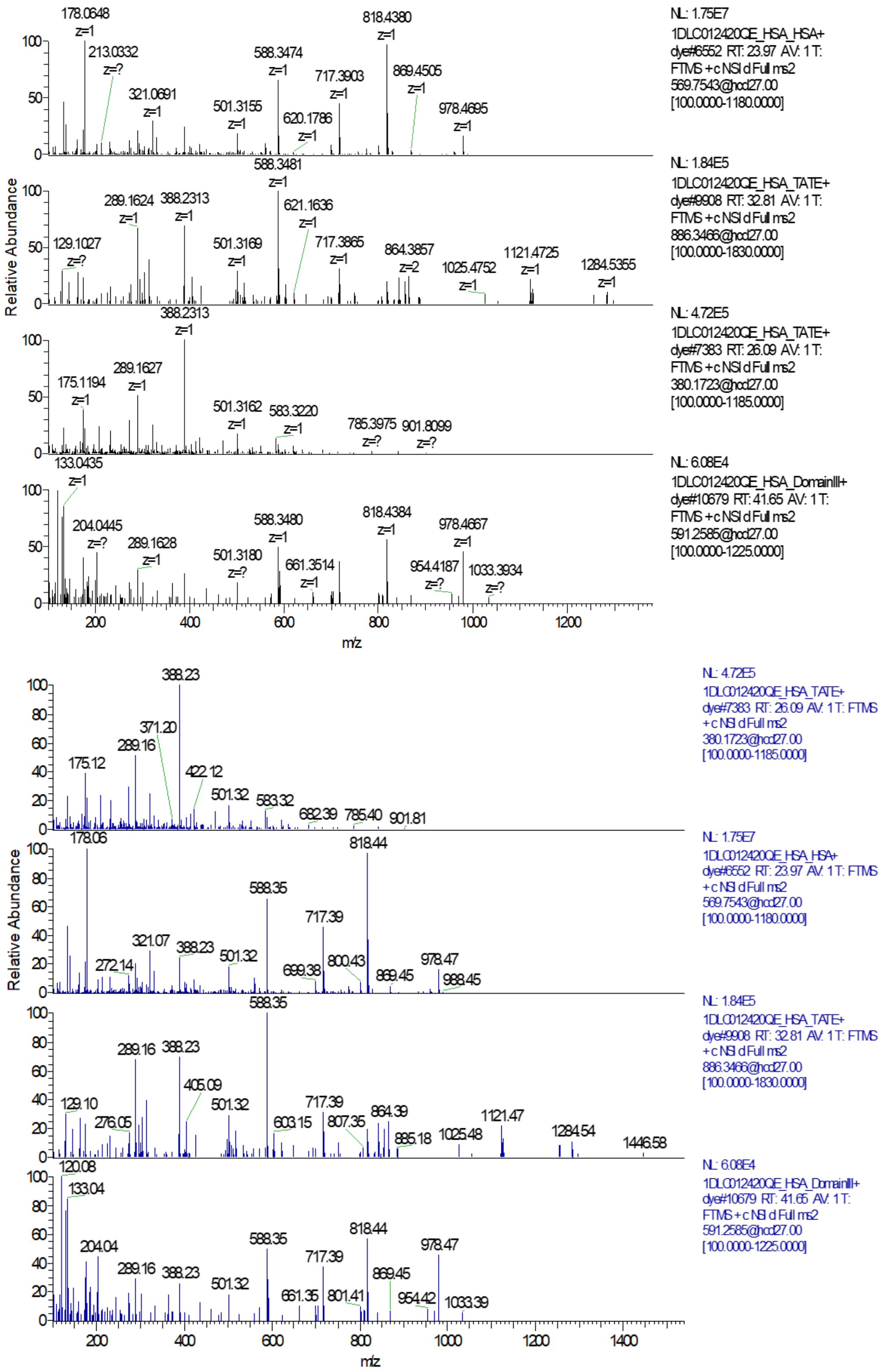

Supplementary Fig. 13. **The exact binding residue on the sequence of TESLVNR.** The backup spectra were analyzed.

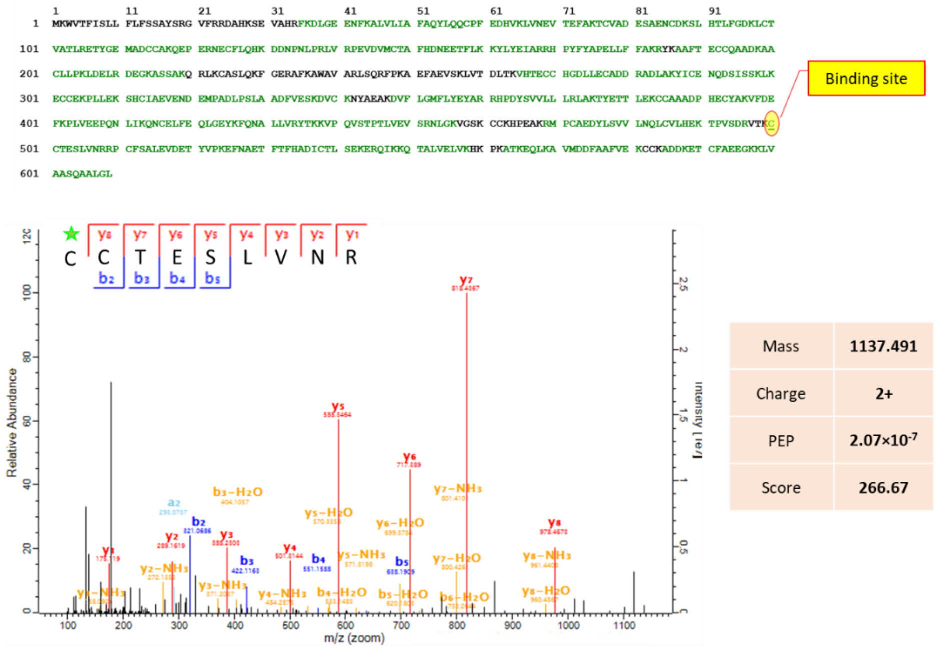

Supplementary Fig. 14. Shotgun proteomics analysis of dye-labeled HSA. It revealed that Cys476 also contributes to the formation of the covalent bond. MS/MS spectra of the double charged tryptic peptide containing the dye-modified cysteine (five-pointed star indicated) amino acid. The complete y series ions (y1–y8) were acquired using an Orbitrap-based mass analyzer (<0.5 ppm). Posterior error probability (PEP) and Andromeda scores are presented in the right panel.

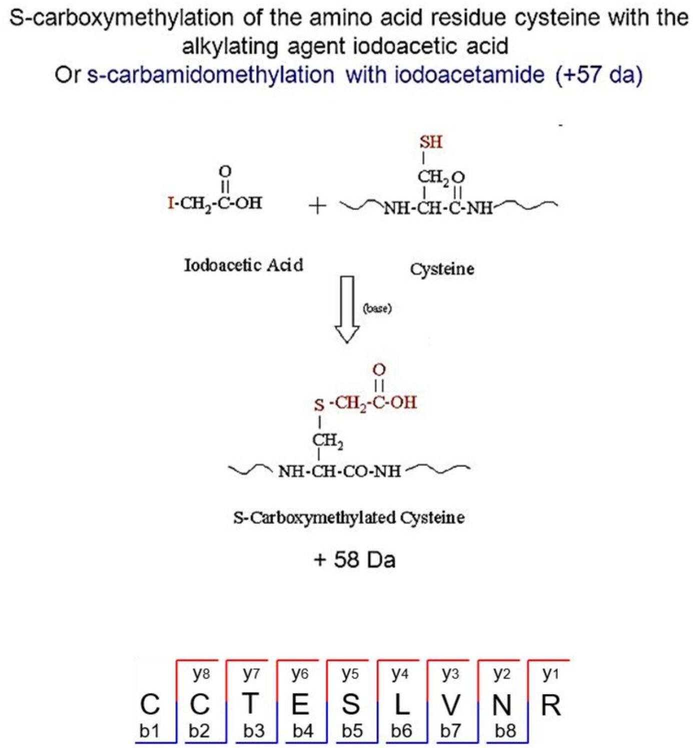

Supplementary Fig. 15. The procedure of carbamidomethylation (Car) for SH- groups. The DIII and HSA solutions were first treated with Car, then the free SH- groups were designedly carbamidomethylated, finally, the carbamidomethylated thiol groups were detected.

a Free ICG

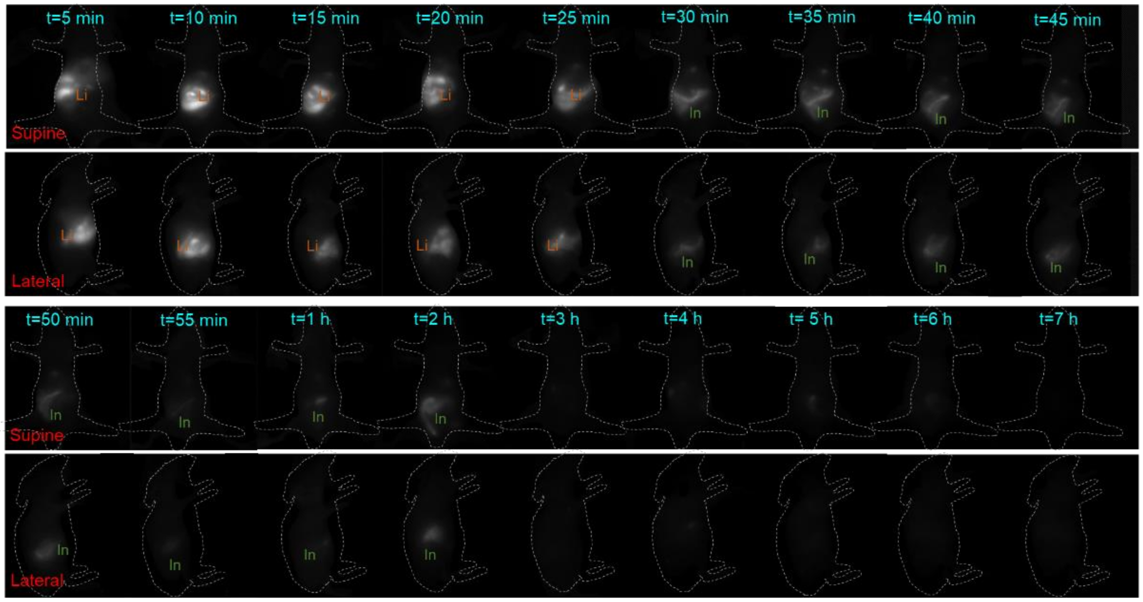

b IR-783@DIII

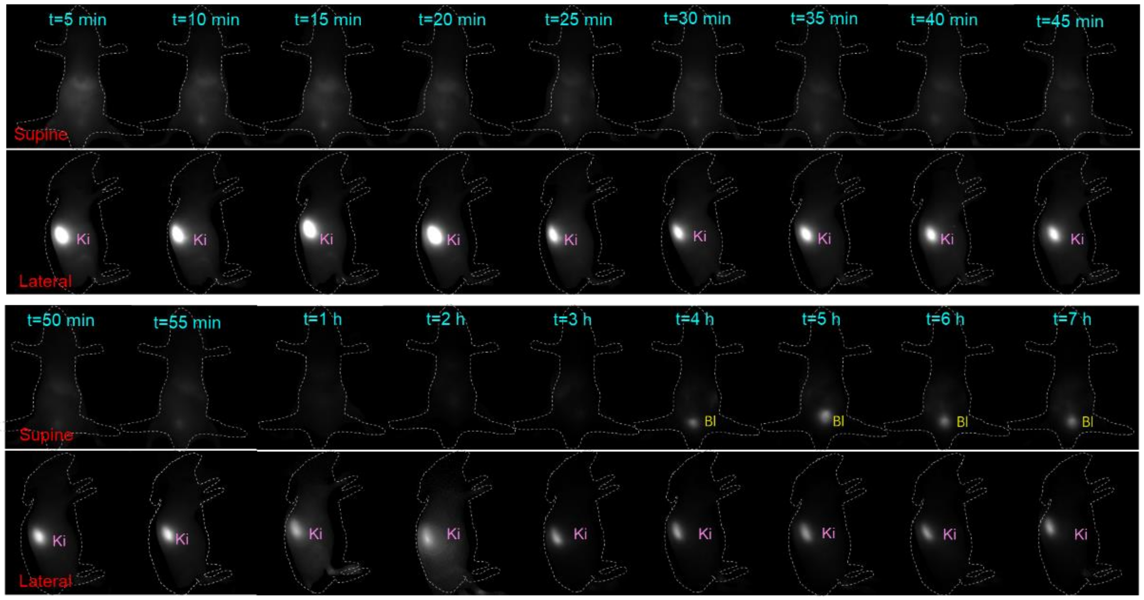

Supplementary Fig. 16. Comparison of the detailed clearance pathway of clinically-used ICG and IR-783@DIII in healthy mice through dynamic NIR-II images. The ICG showed fast hepatobiliary clearance and IR-783@DIII showed renal clearance. These results and the results in Fig. 4 indicated that the DIII totally changed the pharmacokinetic profile of Cl- containing cyanine dyes.

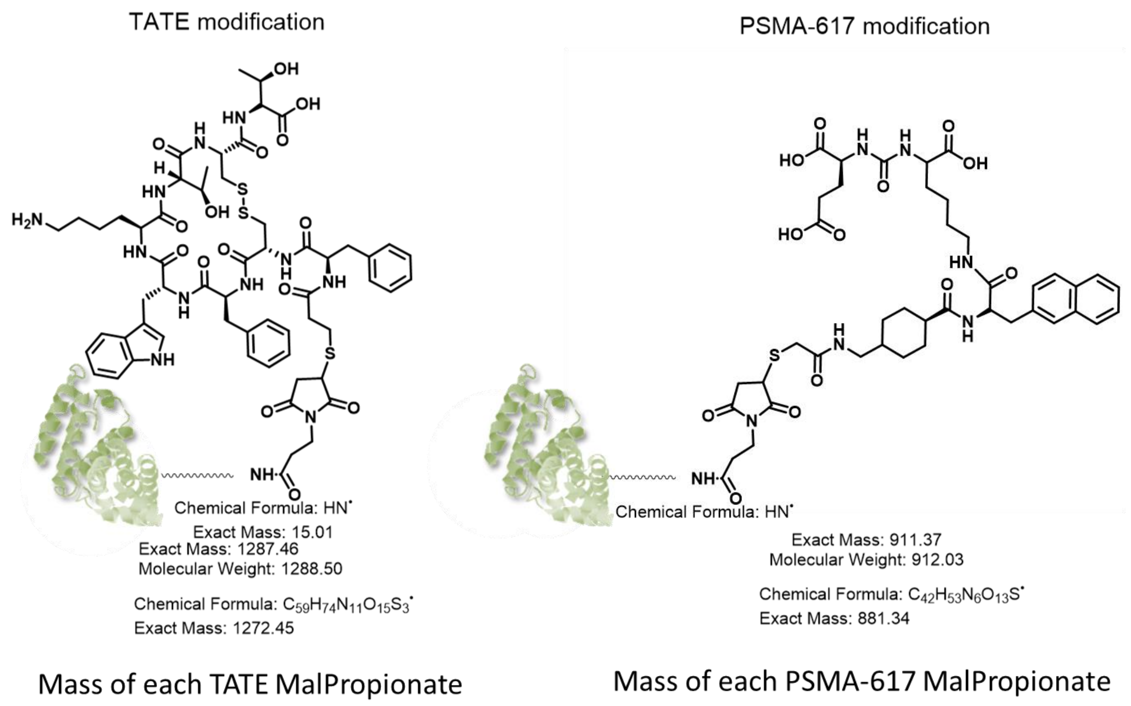

Supplementary Fig. 17. The conjugation strategy for dye@albumin (albumin sub-domain) and desired peptides. The exact mass of each TATE and PSMA-617 was listed for LC-MS analysis.

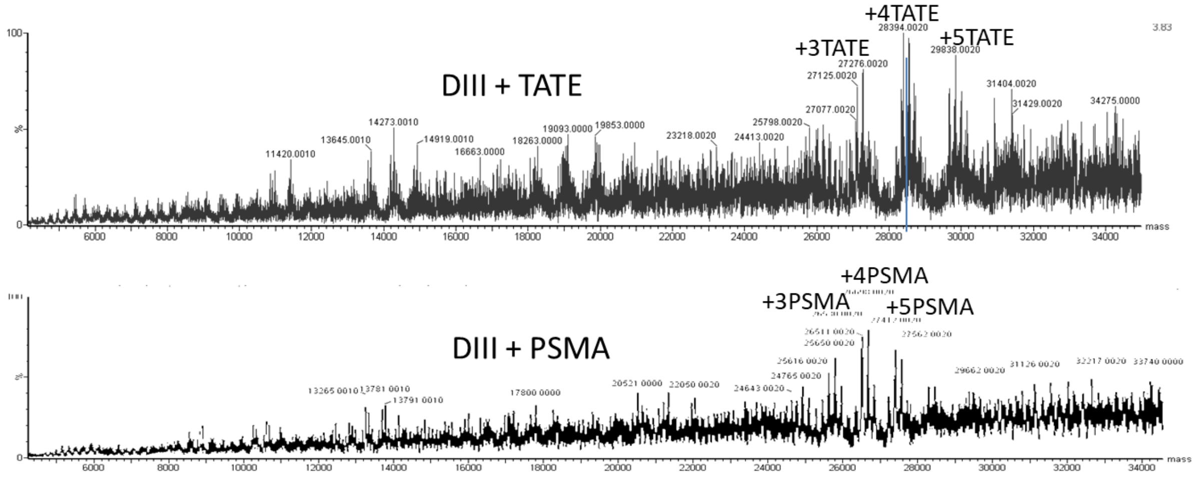

Supplementary Fig. 18. The successful conjugation of dye@DIII and desired peptide or molecule. The conjugation of dye@DIII and TATE or PSMA-617 (PSMA) molecule was quantificationally confirmed by superhigh-resolution LC-MS with both measured molecular weights equal to their theoretical prediction.

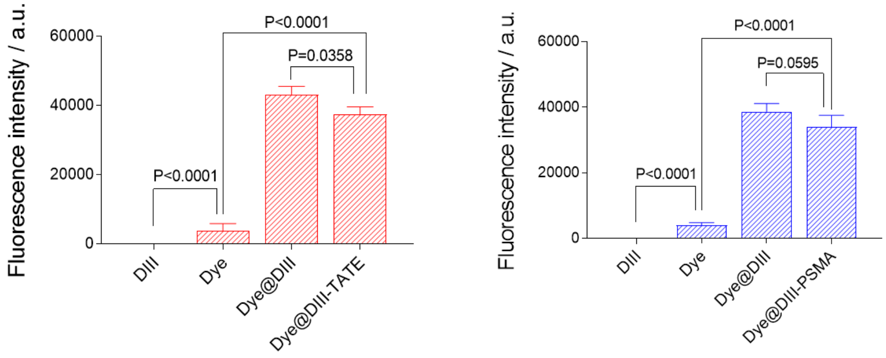

Supplementary Fig. 19: Quantification of fluorescence intensity from different samples under NIR-II imaging (n = 3)**.** Data are presented as mean ± s.d. of three independent experiments, analyzed by unpaired student T-test (two-sided).

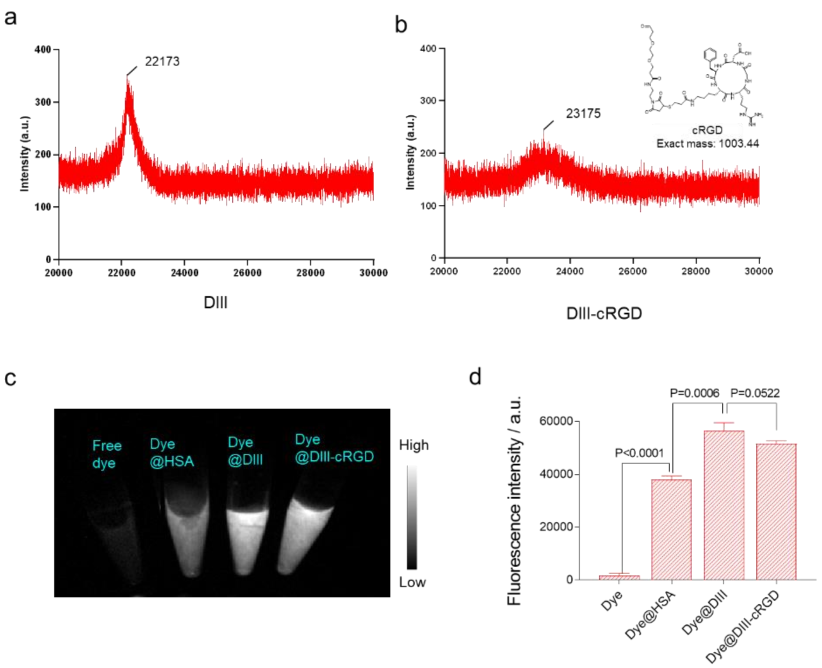

Supplementary Fig. 20: Dye@DIII-cRGD was synthesized and evaluated. a-b) Successful synthesis of DIII-cRGD was confirmed with Matrix-assisted laser desorption ionization time-of-flight mass spectroscopy (MALDI-TOF MS). c) Comparison of fluorescence intensity of different samples. d) Quantification of fluorescence intensity from different samples (n = 3). Data are presented as mean ± s.d. of three independent experiments, analyzed by unpaired student T-test (two-sided).

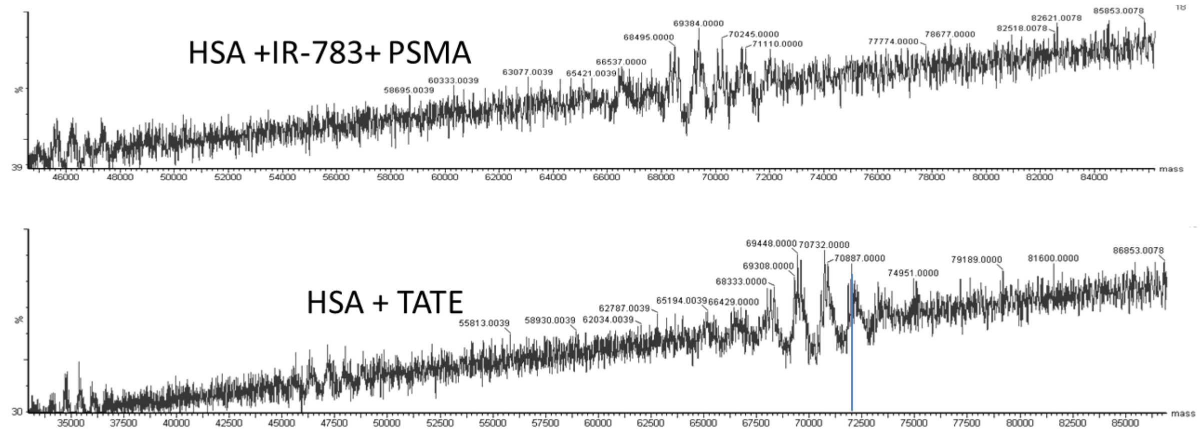

Supplementary Fig. 21. The successful conjugation of dye@HSA and selected peptides (TATE and PSMA). The conjugation of dye@HSA and TATE or PSMA-617 (PSMA) molecule was confirmed by the superhigh-resolution LC-MS with calculated molecular weights.

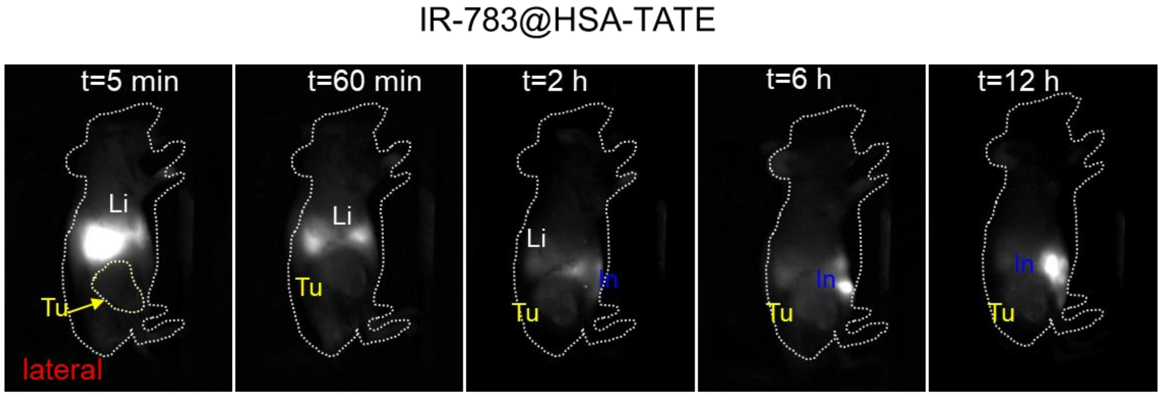

Supplementary Fig. 22. The tumor accumulation ability of IR-783@HSA-TATE probe in AR42J tumor xenografts. Selected time points of NIR-II imaging of AR42J tumor-bearing mouse after an intravenous injection of IR-783@HSA-TATE probe. The injection dosage and imaging conditions were equal to that of IR-783@DIII-TATE administered cohort of tumor xenografts. IR-783@DIII-TATE shows greatly improved molecular imaging ability in terms of higher tumor-to-muscle ratio and suppressive liver/intestine signals.

IR-783@DIIIa

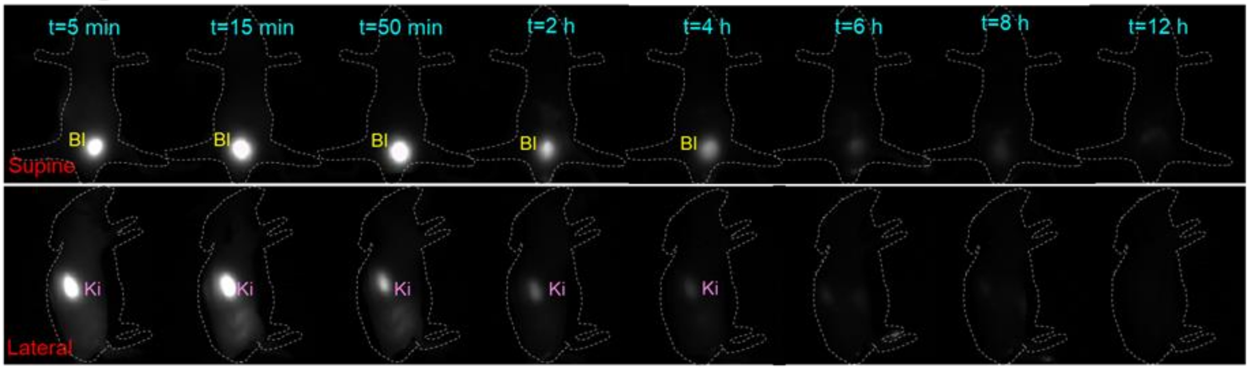

Supplementary Fig. 23. The pharmacokinetic profile IR-783@DIIIa in healthy mice. IR-783@DIIIa showed quick renal clearance.

Note: Compared with than IR-783@DIII (Supplementary Fig. 16b), IR-783@DIIIa showed more accumulation in bladder at early time point, and it cleared faster than IR-783@DIII (more accumulation in kidney but less in bladder at early time point).

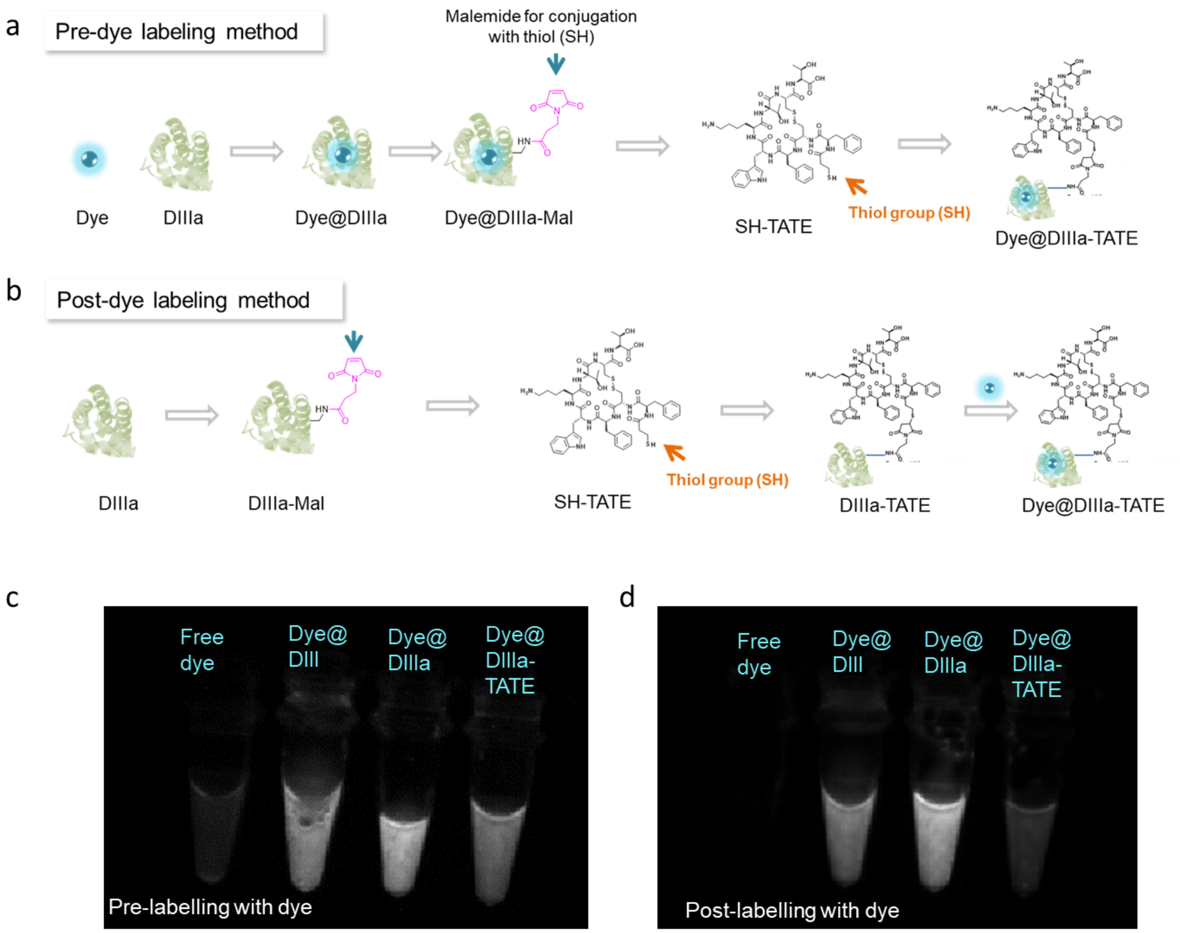

Supplementary Fig. 24. The method of pre- and post-dye labeling. a) Schematic process of pre-labeling for dye@DIIIa-TATE. b) Schematic process of post-labeling for dye@DIIIa-TATE. c,d) Brightness comparison of dye@DIIIa-TATE with free dye, dye@DIII, dye@IIIa (with the same molar concentrations) through independent experimental sets of pre- and post- labeling approaches. Both of these approaches manufactured much brighter and more stable complex compared with the post-labeling one, even the labeling/binding efficiency was theoretically comparable.

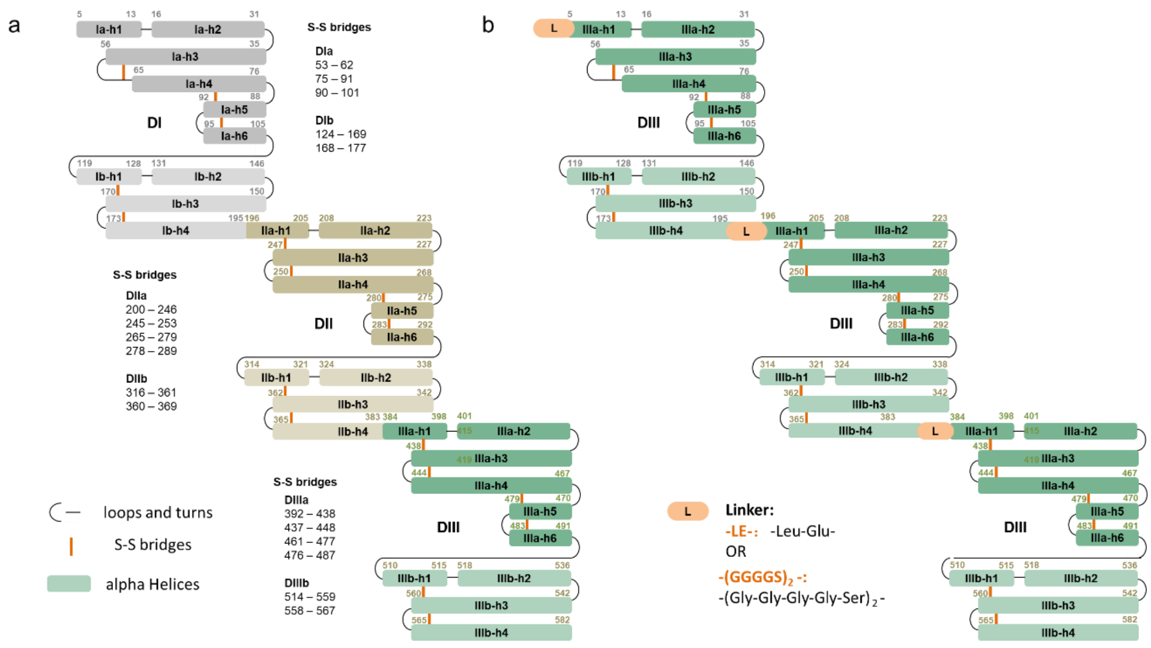

Supplementary Fig. 25. Schematic diagram of designed TDIII structure (b). a) represents the structure of wide type albumin. To achieve an albumin-like in vivo pharmacokinetic behavior, several natural linkers were screened to keep the nature properties of fused protein, e.g., leucine-glutamic acid (Leu-Glu) and (glycine-glycine-glycine-glycine-serine)$_2$ (-[Gly-Gly-Gly-Gly-Ser]$_2$-)

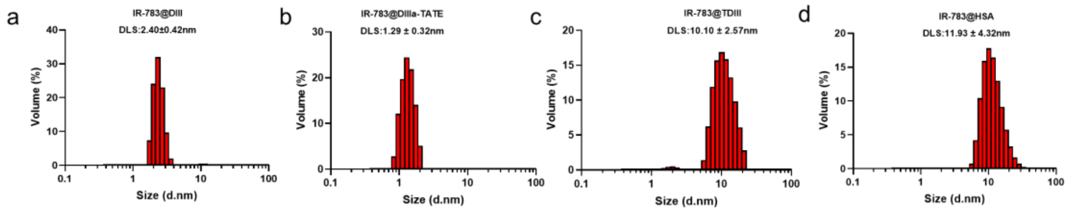

Supplementary Fig. 26. Hydrodynamic sizes of IR-783@DIII and IR-783@DIIIa-TATE, IR-783@TDIII, and IR-783@HSA.

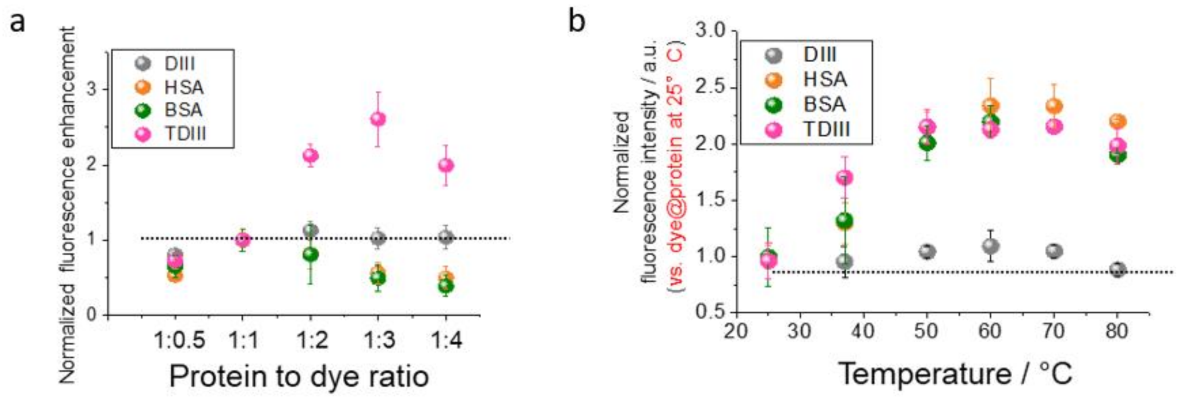

Supplementary Fig. 27. Normalized fluorescence enhancement of dye and proteins complexes. a) Fluorescence enhancement of IR-783 complexed with DIII, HSA, BSA, or TDIII at various molar ratios normalized to the fluorescence intensity of each sample at the molar ratio 1:1 (n = 3). b) Fluorescence intensity of IR-783 complexed with DIII, HSA, BSA, or TDIII at the molar ratio 1:1 at various temperatures normalized to the results at 25 °C (n = 3).

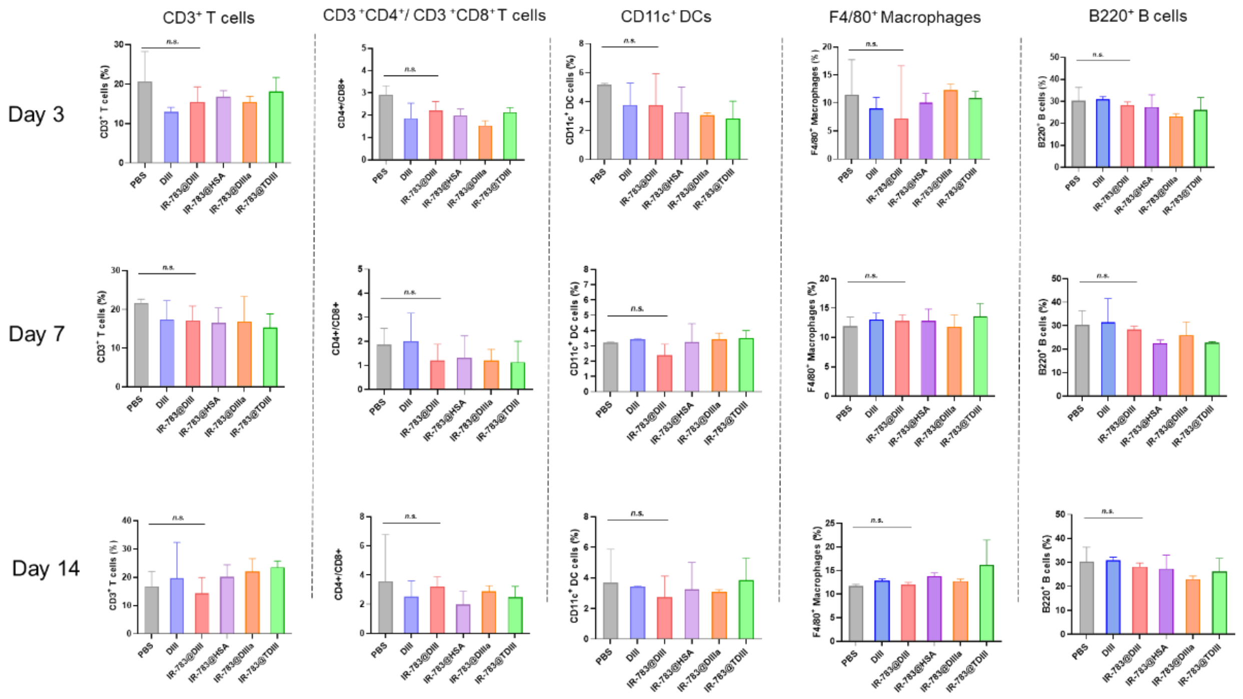

Supplementary Fig. 28. Peripheral blood cells analysis using flowcytometry. The subtypes of lymphatic cells were obtained at 3, 7, and 14 days from mice (n=3 per group) with intravenous injection of PBS, DIII, IR-783@DIII, IR-783@HSA, IR-783@DIIIa, and IR-783@TDIII, respectively. The percentage of CD3+ T cells, CD3+CD4+ and CD3+CD8+ T, CD11c+ DC cells, F4/80+ macrophages, and B220+B cells were analyzed through using flow cytometry. Data are shown as the mean ± s.e.m, analyzed by one-way ANOVA. n.s.: no significant difference (p>0.05).

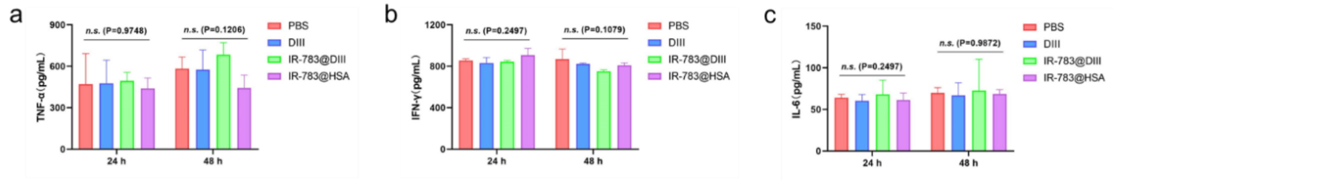

Supplementary Fig. 29. Levels of cytokines in sera from mice isolated at different time points. At the indicated time points, peripheral blood harvested were used to analyze the amount of cytokine, including TNF-α (a), IFN-λ (b) and IL-6 (c). Data are shown as the mean ± s.e.m, analyzed by one-way ANOVA. n.s.: no significant difference (p>0.05).

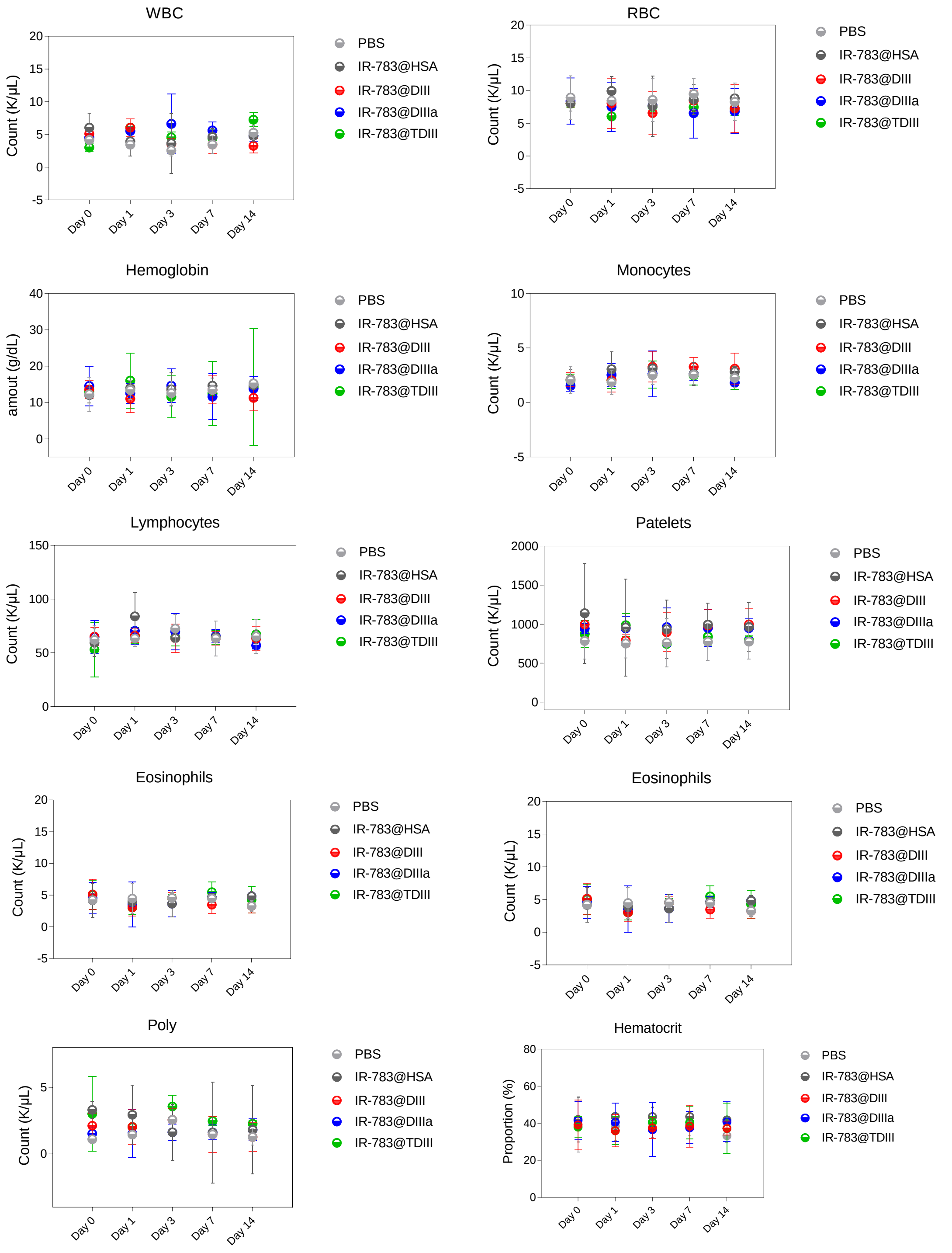

Supplementary Fig. 30. Animal hematology test (complete blood counts) from mice injected with of PBS, DIII, IR-783@DIII, IR-783@HSA, IR-783@DIIIa, and IR-783@TDIII at 24 h post-injection, respectively. Data are shown as the mean ± s.e.m, no significant differences were observed and analyzed by one-way ANOVA, and also, they are in the normal range.

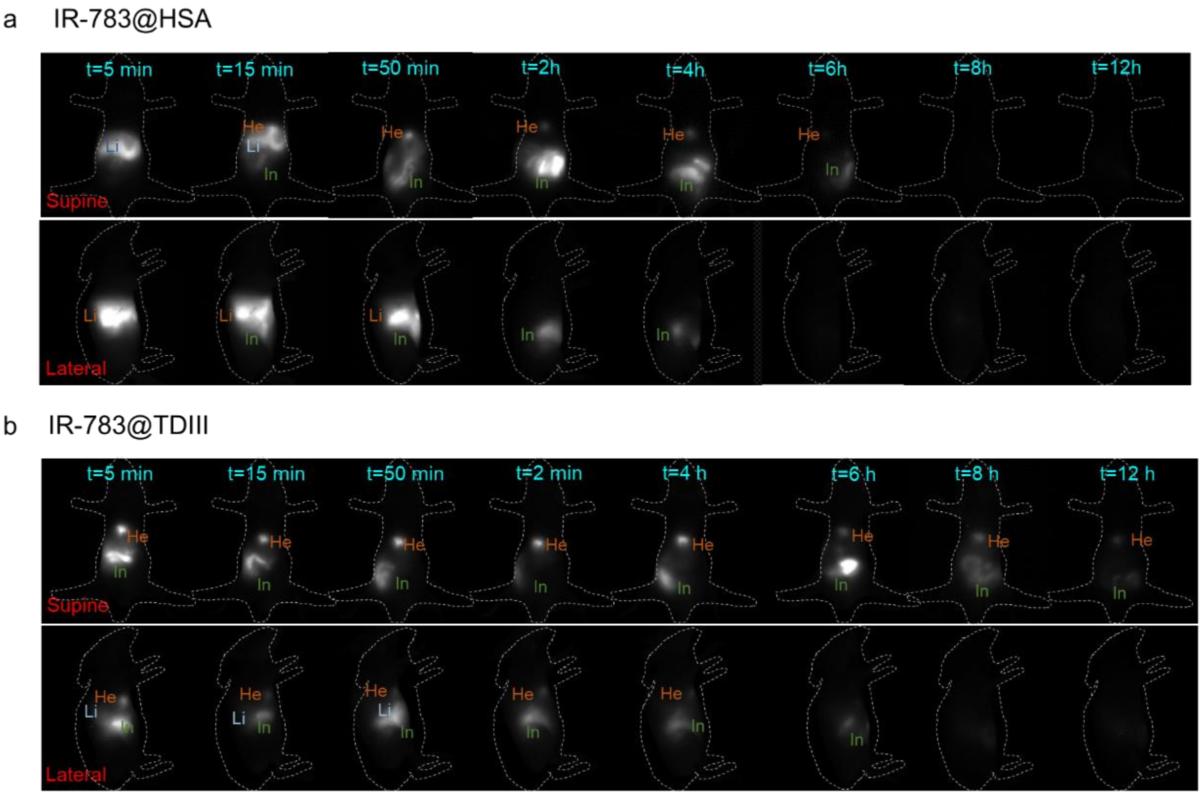

Supplementary Fig. 31. Comparison of pharmacokinetic profile of IR-783@TDIII and IR-783@HSA in healthy mice. Both of them showed long circulation time and hepatobiliary clearance.

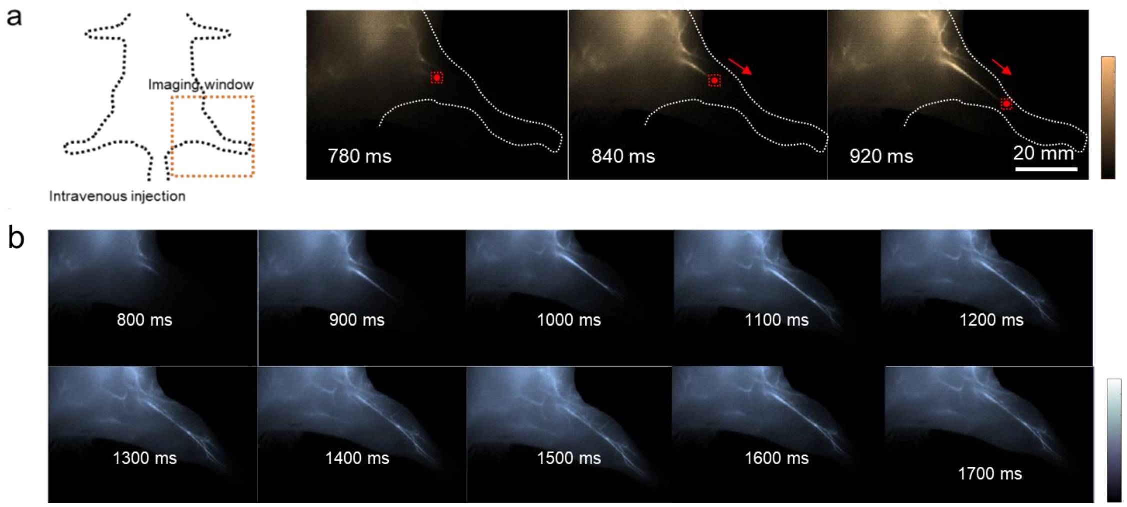

Supplementary Fig. 32. Real-time NIR-II vessel imaging was performed using dye@TDIII probe. a) The migration speed of the fluorophore (blood speed) in the hindlimb vessel was precisely calculated according to the several early time points from video-rate NIR-II imaging of a mouse hindlimb after tail vein injection of dye@TDIII probe. b) Selected longer time points (800 to 1700 millisecond) from the same video-rate NIR-II imaging outlined the blood flow.

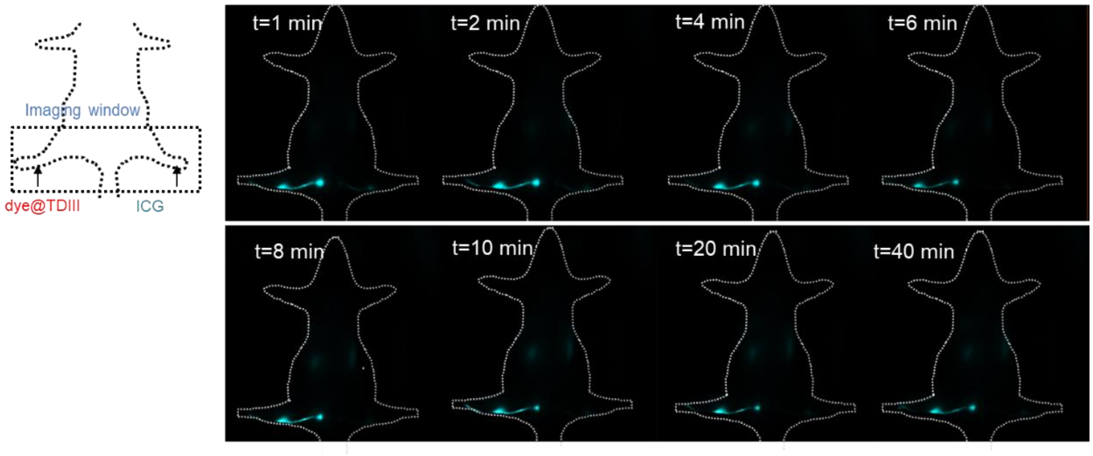

Supplementary Fig. 33. The photostability of dye@TDIII and the clinically used ICG was compared side by side under the continuous laser exposure.

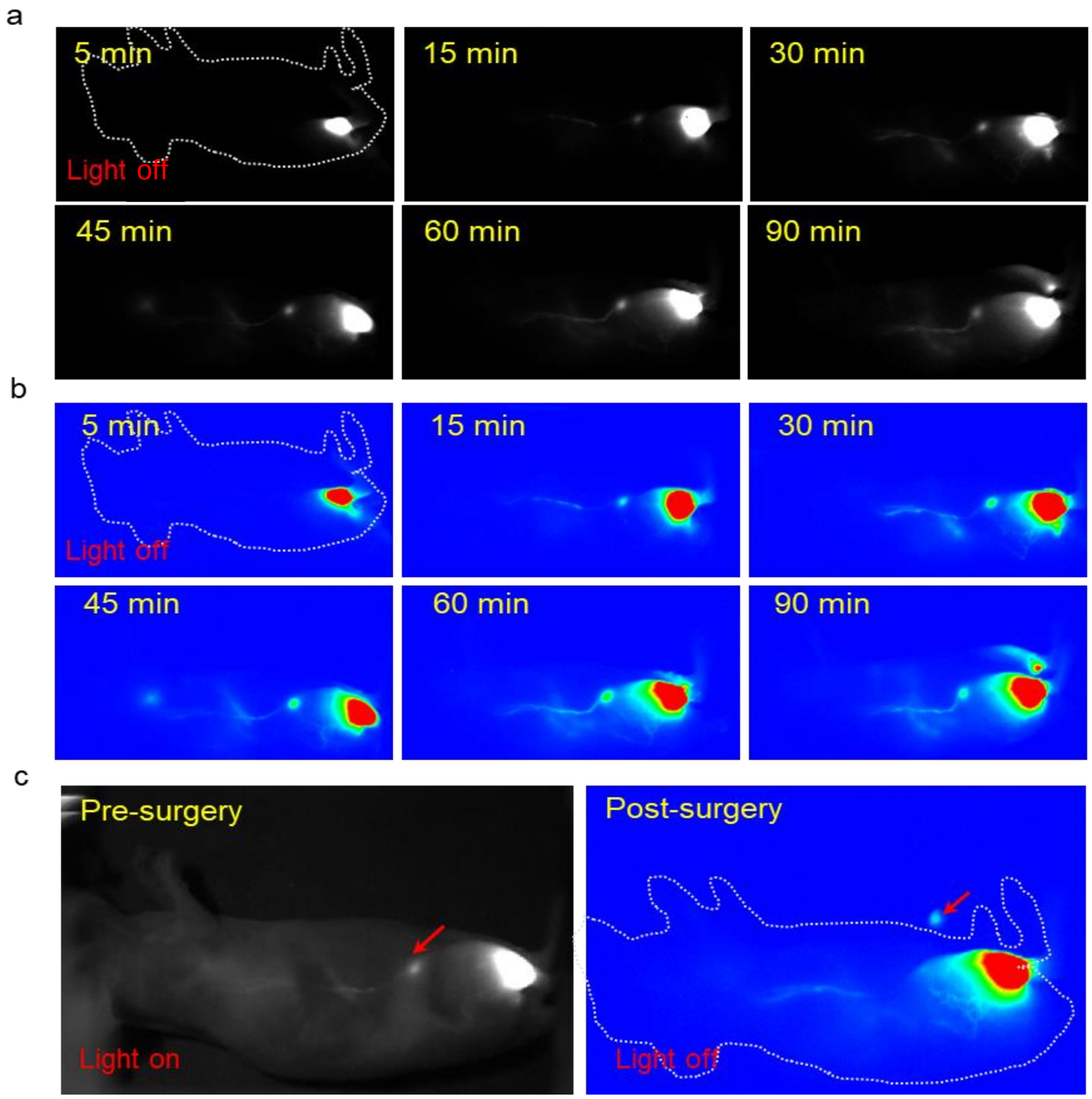

Supplementary Fig. 34. More cases of intraoperative navigation with dye@TDIII probe. a) After intratumor injection of dye@TDIII probe, NIR-II imaging provided high-quality visualization of tumor-adjacent LNs with light on (a) or light off (pseudo-color) (b). c) NIR-II imaging-guided surgery was subsequently performed to accurately excise the tumor-adjacent LNs.
