# Supplementary material for: A genetic engineering strategy for editing near-infrared-II fluorophores
Source: Nat Commun. 2022 May 23;13:2853. doi: 10.1038/s41467-022-30304-9 (PMC9127093; doi:10.1038/s41467-022-30304-9)
Supplement: Supplementary file 2 — Reporting Summary [file 41467_2022_30304_MOESM2_ESM.pdf]

nature portfolio

Corresponding author(s): Xiaoyuan Chen

Last updated by author(s): Apr 2, 2022

Reporting Summary

Nature Portfolio wishes to improve the reproducibility of the work that we publish. This form provides structure for consistency and transparency in reporting. For further information on Nature Portfolio policies, see our Editorial Policies and the Editorial Policy Checklist.

Statistics

For all statistical analyses, confirm that the following items are present in the figure legend, table legend, main text, or Methods section.

n/a	Confirmed	
☐	☒	The exact sample size (n) for each experimental group/condition, given as a discrete number and unit of measurement
☐	☒	A statement on whether measurements were taken from distinct samples or whether the same sample was measured repeatedly
☐	☒	The statistical test(s) used AND whether they are one- or two-sided<br>*Only common tests should be described solely by name; describe more complex techniques in the Methods section.*
☒	☐	A description of all covariates tested
☐	☒	A description of any assumptions or corrections, such as tests of normality and adjustment for multiple comparisons
☐	☒	A full description of the statistical parameters including central tendency (e.g. means) or other basic estimates (e.g. regression coefficient) AND variation (e.g. standard deviation) or associated estimates of uncertainty (e.g. confidence intervals)
☐	☒	For null hypothesis testing, the test statistic (e.g. F, t, r) with confidence intervals, effect sizes, degrees of freedom and P value noted<br>*Give P values as exact values whenever suitable.*
☒	☐	For Bayesian analysis, information on the choice of priors and Markov chain Monte Carlo settings
☒	☐	For hierarchical and complex designs, identification of the appropriate level for tests and full reporting of outcomes
☒	☐	Estimates of effect sizes (e.g. Cohen's d, Pearson's r), indicating how they were calculated

Our web collection on statistics for biologists contains articles on many of the points above.

Software and code

Policy information about availability of computer code

Data collection	UV-Vis absorption spectra were abtained on a Genesys 10S UV-Vis-NIR spectrophotometer (Thermo Scientific , Waltham, MA). Fluorescence spectrophotometry was carried out on a Hitachi F-7000 fluorescence spectrophotometer. Fluorescence microscopy was performed on an Olympus fluorescence microscope. NIR-I imaging was performed on a CRi Maestro in vivo imaging system. NIR-II images were collected on a two-dimensional InGaAs array (Princeton Instruments). Lens and NIR achromats were used from Thorlabs (900 SP/LP, 1000 SP/LP, 1100 SP/LP, 1200 SP/LP, 1300 SP/LP and 200 mm and 75 mm achromats, Thorlabs). Protein fragments were performed on Vivacon filters (30K MWKO, Sartorius, Germany).
Data analysis	ChembioDraw Ultra 13.0/Origin 2018/Graphpad prism 8/Image J (1.52a, java 1.8.0_66)/Gaussian 16 software/Proteome Discoverer platform (version 1.4, Thermo Scientific)/MaxQuant software (version 1.5.1.0)/Sequest HT (version 2.4, Matrix Science)/Perseus software (version 1.5)

For manuscripts utilizing custom algorithms or software that are central to the research but not yet described in published literature, software must be made available to editors and reviewers. We strongly encourage code deposition in a community repository (e.g. GitHub). See the Nature Portfolio guidelines for submitting code & software for further information.

Data

Policy information about availability of data

All manuscripts must include a data availability statement. This statement should provide the following information, where applicable:

- Accession codes, unique identifiers, or web links for publicly available datasets
- A description of any restrictions on data availability
- For clinical datasets or third party data, please ensure that the statement adheres to our policy

All data supporting the findings of this study are available within the article and its Supplementary Information files or from the corresponding authors on

reasonable request. Source data are provided as a Source Data file.

Field-specific reporting

Please select the one below that is the best fit for your research. If you are not sure, read the appropriate sections before making your selection.

- [x] Life sciences
- [ ] Behavioural & social sciences
- [ ] Ecological, evolutionary & environmental sciences

For a reference copy of the document with all sections, see nature.com/documents/nr-reporting-summary-flat.pdf

Life sciences study design

All studies must disclose on these points even when the disclosure is negative.

Sample size	Sample sizes were not chosen based on statistical method. Samples sized was chosen as large as possible to be sufficient to obtain statistics (n= 5-25). For in vitro experiments, samples were prepared and measured a minimum of three twice. For in vivo experiments, each group has 5 biologically independent mice. All data are reported as mean ± standard deviation (s.d.) or Standard Error of Mean (s.e.m.) from at least three independent runs. Significant differences in the mean values were evaluated by Student's unpaired t-test (two-sided) or one-way ANOVA.
Data exclusions	No data was excluded.
Replication	Every experiment at replication was successful. All experiments were performed a minimum of three replicates in independent experiments with similar results.
Randomization	Randomly grouped
Blinding	The investigators were blinded to group allocation during data collection and analysis.

Reporting for specific materials, systems and methods

We require information from authors about some types of materials, experimental systems and methods used in many studies. Here, indicate whether each material, system or method listed is relevant to your study. If you are not sure if a list item applies to your research, read the appropriate section before selecting a response.

Materials & experimental systems

n/a	Involved in the study	
[ ]	[x]	Antibodies
[ ]	[x]	Eukaryotic cell lines
[x]	[ ]	Palaeontology and archaeology
[ ]	[x]	Animals and other organisms
[x]	[ ]	Human research participants
[x]	[ ]	Clinical data
[x]	[ ]	Dual use research of concern

Methods

n/a	Involved in the study	
[x]	[ ]	ChIP-seq
[ ]	[x]	Flow cytometry
[x]	[ ]	MRI-based neuroimaging

Antibodies

Antibodies used

APC anti-mouse CD3, Biolegend, catalog n. 100236
FITC anti-mouse CD4, Biolegend, catalog n. 100405
Pacific Blue™ anti-mouse CD8a, Biolegend, catalog n. 100725
APC anti-mouse F4/80, Biolegend, catalog n. 123115
FITC anti-mouse CD11c, Biolegend, catalog n. 117305
Brilliant Violet 421™ anti-mouse/human CD45R/B220, Biolegend, catalog n. 103239

Validation

The antibodies are verified by the supplier. The quality test data was showed on the manufacturers' websites as following.
APC anti-mouse CD3, Biolegend, catalog n. 100236
https://www.biolegend.com/en-us/products/apc-anti-mouse-cd3-antibody-8055
FITC anti-mouse CD4, Biolegend, catalog n. 100405
https://www.biolegend.com/en-us/products/fitc-anti-mouse-cd4-antibody-248
Pacific Blue™ anti-mouse CD8a, Biolegend, catalog n. 100725
https://www.biolegend.com/en-us/products/pacific-blue-anti-mouse-cd8a-antibody-2856
APC anti-mouse F4/80, Biolegend, catalog n. 123115
https://www.biolegend.com/en-us/products/apc-anti-mouse-f4-80-antibody-4071
FITC anti-mouse CD11c, Biolegend, catalog n. 117305

https://www.biolegend.com/en-us/products/fitc-anti-mouse-cd11c-antibody-1815
Brilliant Violet 421™ anti-mouse/human CD45R/B220, Biolegend, catalog n. 103239
https://www.biolegend.com/en-us/products/brilliant-violet-421-anti-mouse-human-cd45r-b220-antibody-7158

Eukaryotic cell lines

Policy information about cell lines

Cell line source(s)	AR42J, PC3, and 4T1-fluc cell lines were purchased from ATCC. PC3-PIP were obtained from NIBIB/NIH.
Authentication	The cells were only authenticated by morphology
Mycoplasma contamination	Negative for mycoplasma
Commonly misidentified lines (See ICLAC register)	No misidentified lines

Animals and other organisms

Policy information about studies involving animals; ARRIVE guidelines recommended for reporting animal research

Laboratory animals	Nude, C57BL/6j, and BALB/C (female, 6-8 weeks) were purchased from Jackson's Laboratory (Bar Harbor, ME). The animals were hosted in equipped animal facility with temperature at 68-79 F and humidity at 30%-70%, under the same dark/light cycle (12:12).
Wild animals	No wild animals were used
Field-collected samples	No filed-collection
Ethics oversight	All animal experiments were performed under a National Institutes of Health Animal Care and Use Committee (NIHACUC) approved protocol.

Note that full information on the approval of the study protocol must also be provided in the manuscript.

Flow Cytometry

Plots

Confirm that:

- [x] The axis labels state the marker and fluorochrome used (e.g. CD4-FITC).
- [x] The axis scales are clearly visible. Include numbers along axes only for bottom left plot of group (a 'group' is an analysis of identical markers).
- [x] All plots are contour plots with outliers or pseudocolor plots.
- [x] A numerical value for number of cells or percentage (with statistics) is provided.

Methodology

Sample preparation	Female C57BL/6 mice (6–8 weeks) were intravenously injected with PBS, DIII, IR-783@DIII, IR-783@HSA, IR-783@DIIIa, and IR-783@TDIII, respectively. Peripheral blood was collected to analyze subtypes of lymphatic cells. Briefly, blood was collected from the treated mice on day 3, day 7 and day 14. Blood cells were enriched by centrifugation. Red blood cells were lysed using ACK lysis buffer for 10min at room temperature. Then, cells were washed twice in PBS and stained with the indicated antibodies for 15min. After that, cells were washed with FCS buffer (PBS buffer with 0.1% FBS), and resuspended for flow cytometric analysis. Flow cytometry was conducted on a Beckman CytoFlex S flow cytometer. Data were analyzed using FlowJo V10 version.
Instrument	Beckman CytoFlex S
Software	FlowJo_V10
Cell population abundance	The purity of post-sort fractions measured by the software. The fractions were around 50-70%.
Gating strategy	A forward-scatter/side-scatter (FSC/SSC) was used to gate on cells.

- [x] Tick this box to confirm that a figure exemplifying the gating strategy is provided in the Supplementary Information.
