# Supplementary material for: A genetic engineering strategy for editing near-infrared-II fluorophores
Source: Nat Commun. 2022 May 23;13:2853. doi: 10.1038/s41467-022-30304-9 (PMC9127093; doi:10.1038/s41467-022-30304-9)
Supplement: Supplementary file 3 — Description of Additional Supplementary Files [file 41467_2022_30304_MOESM3_ESM.pdf]

Title: Supplementary Movie 1:
Description: Real-time NIR-II imaging of vessels using IR783@TDIII.

Title: Supplementary Movie 2:
Description: Real-time high-magnification (2.5×) NIR-II imaging was performed in mice with shaved heads.

Title: Supplementary Movie 3:
Description: Real-time high-resolution imaging and detection of congenital lymphedema
